# Protective mechanisms of melatonin against selenium toxicity in *Brassica napus*: insights into physiological traits, thiol biosynthesis and antioxidant machinery

**DOI:** 10.1186/s12870-019-2110-6

**Published:** 2019-11-21

**Authors:** Zaid Ulhassan, Qian Huang, Rafaqat Ali Gill, Skhawat Ali, Theodore Mulembo Mwamba, Basharat Ali, Faiza Hina, Weijun Zhou

**Affiliations:** 1 Institute of Crop Science, Ministry of Agriculture and Rural Affairs Key Laboratory of Spectroscopy Sensing, Zhejiang University, Hangzhou, 310058 China; 2 Oil Crops Research Institute, Chinese Academy of Agricultural Sciences, Wuhan, 430062 China; 3 Department of Agronomy, University of Agriculture, Faisalabad, 38040 Pakistan; 4 Lab of Systematic & Evolutionary Botany and Biodiversity, College of Life Science, Zhejiang University, Hangzhou, 310058 China

**Keywords:** Antioxidants, Oilseed rape, Osmolytes, Oxidative stress, Plant growth regulator, Selenium, Thiols

## Abstract

**Background:**

The ubiquitous signaling molecule melatonin (*N*-acetyl-5-methoxytryptamine) (MT) plays vital roles in plant development and stress tolerance. Selenium (Se) may be phytotoxic at high concentrations. Interactions between MT and Se (IV) stress in higher plants are poorly understood. The aim of this study was to evaluate the defensive roles of exogenous MT (0 μM, 50 μM, and 100 μM) against Se (IV) (0 μM, 50 μM, 100 μM, and 200 μM) stress based on the physiological and biochemical properties, thiol biosynthesis, and antioxidant system of *Brassica napus* plants subjected to these treatments.

**Results:**

Se (IV) stress inhibited *B. napus* growth and biomass accumulation, reduced pigment content, and lowered net photosynthetic rate (*P*_*n*_) and PSII photochemical efficiency (*Fv/Fm*) in a dose-dependent manner. All of the aforementioned responses were effectively alleviated by exogenous MT treatment. Exogenous MT mitigated oxidative damage and lipid peroxidation and protected the plasma membranes from Se toxicity by reducing Se-induced reactive oxygen species (ROS) accumulation. MT also alleviated osmotic stress by restoring foliar water and sugar levels. Relative to standalone Se treatment, the combination of MT and Se upregulated the ROS-detoxifying enzymes SOD, APX, GR, and CAT, increased proline, free amino acids, and the thiol components GSH, GSSG, GSH/GSSG, NPTs, PCs, and cys and upregulated the metabolic enzymes γ-ECS, GST, and PCS. Therefore, MT application attenuates Se-induce oxidative damage in plants. MT promotes the accumulation of chelating agents in the roots, detoxifies Se there, and impedes its further translocation to the leaves.

**Conclusions:**

Exogenous MT improves the physiological traits, antioxidant system, and thiol ligand biosynthesis in *B. napus* subjected to Se stress primarily by enhancing Se detoxification and sequestration especially at the root level. Our results reveal better understanding of Se-phytotoxicity and Se-stress alleviation by the adequate supply of MT. The mechanisms of MT-induced plant tolerance to Se stress have potential implications in developing novel strategies for safe crop production in Se-rich soils.

**Graphical abstract:**

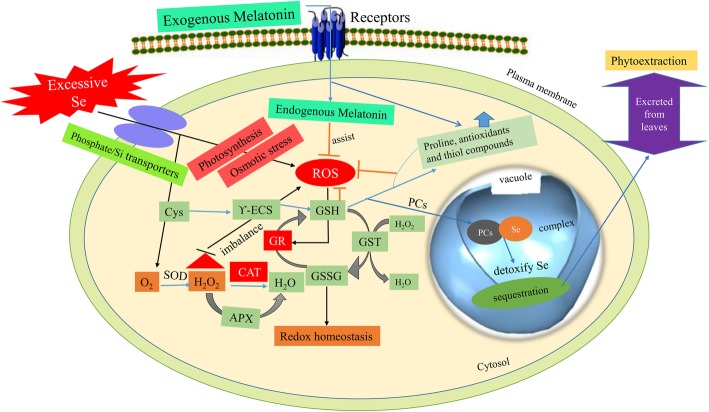

## Highlights

- ➣ Excessive Se inhibits the plant growth, biomass accumulation and impairs photosynthesis
- ➣ Se causes osmotic stress and modulates the thiol metabolism
- ➣ Se induces oxidative injuries by desynchronizing the ROS-detoxifying enzyme activities
- ➣ Exogenous MT protects the physio-biochemical traits by scavenging Se-oxidative damages
- ➣ MT enhances plant tolerance by inducing thiols accumulation to sequester Se in roots.

## Background

The naturally occurring metalloid selenium (Se) is an essential micronutrient/trace element for human and certain animals. However, its effect and importance in plants remain controversial [1]. The essentiality and phytotoxicity of Se may depend on dose, speciation, and target species [2]. Over the past few decades, Se levels have been rising in agricultural soils and could be toxic to plants, humans, and animals [3]. Fossil fuel combustion, mining, irrigation, and industrial discharge are the main sources of large-scale Se pollution [4]. Soil selenium content normally ranges from 0.01–2 mg kg^− 1^. However, in certain regions such as Hubei Province, China, soil Se levels are excessive (> 10 mg kg^− 1^) [5]. Selenite (IV) and selenate (VI) are the mains forms of Se available for plant uptake in soils. While, selenite is transported by phosphate transporters and selenate is mediated by sulfate transporters in different plants [6]. At very high concentrations, both Se-forms are phytotoxic. Nevertheless, Se (IV) is more injurious to plants than Se (VI) and is problematic for farmers [6, 7]. Plants grown in Se-contaminated soils present with chlorosis and stunted growth [8]. Se overdose may perturb photosynthesis, induce reactive oxygen species (ROS) production, and damage plasma membranes by promoting lipid peroxidation [9–11]. In response to oxidative stress, plants produce antioxidant enzymes such as superoxide dismutase (SOD), peroxidase (POD), catalase (CAT), ascorbate peroxidase (APX), and glutathione reductase (GR). Plants also produce thiol ligands such as non-protein thiols (NPTs), cysteine (cys), reduced glutathione (GSH), oxidized glutathione (GSSG), and phytochelatins (PCs) to chelate and detoxify metals and metalloids [12–14].

Melatonin (*N*-acetyl-5-methoxytryptamine) (MT) is a ubiquitous signal molecule with pleiotropic effects and plays regulatory roles for animals and plants. In animals, MT regulates circadian sleep-wake cycle and seasonal reproduction (not in case of plants). Plant ability to synthesize MT in dual organelles (mitochondria and chloroplast) [15]. In higher plants, MT was first discovered in 1995 [16]. It (MT) performs diverse physiological functions such as plant protection against environmental stresses. For this, plants usually enhance endogenous MT production [17]. Under stress conditions, MT promotes plant growth, delays senescence, and modulates photoperiod responses and root architecture [18]. MT may also protect plants against abiotic stressors such as heat [19], cold [20], salt [21], drought [22] and heavy metals [23]. MT augments plant stress tolerance by inducing the enzymatic detoxification of free radicals and reactive oxygen species (ROS) [24] and by scavenging excess ROS [23–25]. However, the crosstalk between MT and metalloids such as Se (IV) is poorly understood and merits further investigation.

Oilseed rape (*Brassica napus* L.) is widely grown as a source of edible oil. It can resist the phytotoxic effects of chromium [26–28], cadmium [29, 30], cobalt [31, 32], beryllium [33], and selenium [10, 11]. Recent reports suggested that 50 μmol kg^− 1^ exogenous MT applied to *Cyphomandra betacea* [34] and 100 μmol L^− 1^ exogenous MT treatment on *Malachium aquaticum* and *Galinsoga parviflora* [35] alleviated cadmium (Cd) toxicity by improving plant growth, photosynthesis, and antioxidant systems. It was reported that 100 μM MT induced the highest antioxidant, GSH, PC, and Cd sequestration levels of all doses tested on tomato [12]. Interactions between MT and Se were recently reported to mitigate Cd toxicity in tomato plants [36]. However, the roles of MT in attenuating Se (IV) phytotoxicity in higher plants) remain unknown. Thiols such as GSH and PCs have proven chelating, antioxidant, and stress tolerance induction properties in plants. However, the mechanisms of MT-prompted thiol biosynthesis and MT-associated Se (IV) resistance in higher plants have not been fully elucidated.

Here, we investigated the influences of MT on Se (IV) stress in higher plants and attempted to uncover the biochemical mechanisms involved. We proposed that MT may play a defensive role in Se (IV) tolerance and participate in other physiological processes besides chelation and antioxidation. We suggested that the forms and levels of thiols induced by plants in response to selenium stress may serve as biomarkers for MT-facilitated Se (IV) stress responses. The aim of the present study was to elucidate the MT-induced mechanisms affecting the physiological and biochemical properties of *B. napus* tissues and their osmotic metabolites and thiol metabolism under Se (IV) stress. This information may be used to assess and mitigate the risks of contamination in food crops raised on soils with elevated Se (IV) burdens.

## Methods

### Plant materials and experimental design

The seeds of black-seeded cultivar ZS (Zheshuang) 758 of *B. napus* (oilseed rape) were obtained from the College of Agriculture and Biotechnology, Zhejiang University, China. The above cultivar was tested previously [11, 37, 38] as tolerant against different heavy metals/metalloids. The seeds were sterilized and germinated at 25 °C in the dark on filter paper in Petri dishes. Germinated seeds were planted in plastic pots (170 mm × 220 mm) containing peat soil. They were maintained in the greenhouse with the following conditions: light intensity of 400 μmol m^− 2^ s^− 1^, temperature of 16–20 °C and relative humidity of 60%. After the emergence of the fifth leaf, uniform-sized seedlings were picked and shifted into plate holes on plastic pots (five plants per pot) having half-strength Hoagland nutrient solution [39]. The nutrient solution was aerated constantly with the air pump. The composition of Hoagland solution was as follows (in μmol/L): 3000 KNO_3_, 2000 Ca (NO_3_)_2_, 1000 MgSO_4_, 10 KH_2_PO_4_, 12 FeC_6_H_6_O_7_, 500 H_3_BO_3_, 800 ZnSO_4_, 50 MnCl_2_, 300 CuSO_4_, 100 Na_2_MoO_4_. The pH of the solution was maintained at 6.0. Each treatment contains four pots (replicates) and nutrient solution was re-filled after every four days. After an acclimatization period of eight days, Se was supplied as sodium selenite (Na_2_SO_3_) by making the desired concentrations (0 μM, 50 μM, 100 μM, 200 μM) and simultaneously supplied MT (50 μM and 100 μM) into the full-strength Hoagland solution. The treatments used were: (1) control (Ck), (2) 50 μM Se (IV) alone, (3) 100 μM Se (IV) alone, (4) 200 μM Se (IV) alone, (5) 50 μM MT alone, (6) 100 μM MT alone, (7) 50 μM Se (IV) + 50 μM MT, (8) 50 μM Se (IV) + 100 μM MT, (9) 100 μM Se (IV) + 50 μM MT, (10) 100 μM Se (IV) + 100 μM MT, (11) 200 μM Se (IV) + 50 μM MT, and (12) 200 μM Se (IV) + 100 μM MT. The selected treatment concentrations were established on the basis of pre-experimental studies, in which different (lower to higher) levels of Se (IV) as 0 μM, 50 μM, 100 μM, 200 μM, 300 μM, 400 μM and 500 μM of Na_2_SO_3_ and MT (0 μM, 25 μM, 50 μM, 100 μM and 200 μM) were applied. The Se (IV) at 50 μM showed slight injuries on plant growth and significant visible damages were prominent at 100 μM Se (IV). While Se (IV) doses higher than 200 μM were too toxic for plant growth. In case of MT application, plants exhibited optimum response at 50 μM and 100 μM MT under Se (IV) stress conditions. The selection of particular (phosphate/silicon) transporter genes was made on the basis of our pre-experimental findings. In preliminary studies, we performed the expression analysis for phosphate (*OsPT1, OsPT2*, *OsPT4*, *OsPT6*, and *OsPT10*), sulfate (*SulTR1*), and silicon (*Lsi2*) transporter genes to find out the potential candidate gene for selenite uptake in the leaves and roots of *B. napus*. These transporter genes (*OsPT2 and Lsi2)* were selected due to their relatively higher abundance. Usually plants up-regulated the expression of phosphate transporter genes in roots [6, 40, 41]. Therefore, we targeted plant roots for the gene expression of these transporters. The experiment was terminated after fifteen days of Se (IV) and MT (alone and combine) treatments. Then plants were harvested for the physio-biochemical, metabolic and anatomical studies.

### Morphological parameters and relative water content (RWC)

Directly after harvesting, fresh biomass of leaves and roots was measured according to [42]. Then plant samples were oven-dried (70 °C) for 4 h. The measurement of full plant lengths, root and leaf area was done according to [26]. Fully stretched fresh leaves (fourth from the apex) per replicate were used for the determination of RWC as reported by [43–45] with minor adjustments. In details, fresh leaves (without midrib) were weighed directly and floated on the surface of deionized distilled water (DDW) in Petri dishes to soak water for the next 48 h in dark. The sticking water of leaf parts was blotted and turgor weight was noted. After dehydrating these samples at 70 °C for 48 h, dry weights were obtained. RWC was calculated by the below formula:

$$ \mathrm{RWC}=\frac{\mathrm{Fresh}\ \mathrm{weight}-\mathrm{Dry}\ \mathrm{weight}}{\kern0.5em \mathrm{Turgid}\ \mathrm{weight}-\mathrm{Dry}\ \mathrm{weight}}\times 100 $$

### Pigment contents, gas exchange, and chlorophyll fluorescence measurement

The light harvesting pigment contents including chlorophylls (*a*, *b*) and carotenoids were extracted from the upper second fully developed leaves with 96% (v/v) ethanol as reported earlier [11]. Net photosynthetic rate (*Pn*) was recorded using an infrared gas analyzer (IRGA) portable photosynthesis system (Li-Cor 6400, Lincoln, NE, USA) as reported by [32]. For the determination of maximum quantum efficiency of photosystem II (*Fv/Fm*), second fully expanded leaves were first reserved in the dark adaptation for 20 min and then measurement of *Fv/Fm* was carried by an imaging pulse-amplitude-modulated (PAM) fluorimeter (IMAG-MAXI; Heinz Walz, Effeltrich, Germany) [46].

### Extraction and quantification of endogenous se and MT by HPLC-MS

The endogenous Se in plant tissues was extracted by the method as reported earlier [11]. The measurement of endogenous plant MT was carried out with some modifications [47]. Fresh samples (0.5 g) of leaf and root were grounded and homogenized in 5 mL methanol containing 50 ng mL^− 1^ [_2_H^6^]-MT (Toronto Research Chemicals Ltd., Toronto, Ontario, Canada) which was used as internal standard. After shaking the homogenate overnight in the dark at 4 °C and centrifuged at 15,000 g for 10 min. Later after transferring the supernatant into a new tube, the segments were again extracted with 2 mL of methanol and mixed with the fraction of supernatant. For the purification of MT, the supernatant was transferred to the C^18^ solid-phase extraction (SPE) cartridge (Waters, Milford, MA, USA). Then extracted material was rigorously dehydrated under nitrogen. The obtained residue was dissolved in 0.5 mL of methanol (70%) and subjected to HPLC electrospray ionization/MS-MS analysis on an Agilent 6460 triple quad LC/MS with an Agilent-XDB^18^ column (2.1 mm × 150 mm, an Agilent Technologies, Frankfurt, Germany). The recovery rate was estimated by the quantification of [_2_H^6^]-MT as an internal standard [48].

### Soluble sugar, free amino acids and proline contents

The method reported by [49] was adapted for the estimation of soluble sugar contents. The estimation of total free amino acids and proline contents was done according to the methods used by [50, 51] respectively.

### Quantification of MDA, ROS, relative electrolyte leakage (REL) and histochemical identification of H_2_O_2_ and O_2_^•–^ as stress markers

The contents of H_2_O_2_, O_2_^•–^ and MDA were determined by following the method described by [32]. Little changes were adopted in TBA method used for MDA determination. Fresh samples (0.2 g) were homogenized, extracted in 10 mL of 0.25% TBA made in 10% trichloroacetic acid (TCA). Then extracted material was heated for 30 min at 95 °C and ice-cooled to terminate the reaction. After centrifuging the cooled mixture at 10,000 g for 10 min, the absorbance of the supernatant was measured at 532 nm. Non-specific turbidity was corrected by subtracting the absorbance values taken at 600 nm and MDA levels were calculated using an extinction coefficient of 155 mM^− 1^ cm^− 1^. The accumulation of H_2_O_2_ and O_2_^•–^ in *B. napus* roots was identified by staining with 3, 3-diaminobenzidine (DAB) and nitroblue tetrazolium (NBT) as done by [38]. For REL, root (0.1 g) sections were shaken for 30 min in deionized water and, then the conductivity of the bathing medium (EC1) was measured. Again, the samples were boiled for 15 min and second conductivity was measured (EC2) [52]. Total electrical conductivity was determined by using the below formula.

$$ \mathrm{REL}\ \left(\%\right)=\left(\frac{\mathrm{EC}1}{\ \mathrm{EC}2}\right)\times 100 $$

### ROS-detoxifying enzymes

For enzymes analysis, leaf and root samples (0.5 g each) were homogenized in 50 mM KH_2_PO_4_ buffer (pH 7.8) and centrifuged at 10,000 g (Eppendorf AG, model 2231, Hamburg, Germany). The floating liquid (above precipitate) was taken for the analysis of subsequent enzyme activities. Total superoxide dismutase (SOD, EC 1.15.1.1) was determined by following the method of [53]. Peroxidase (POD, EC.11.1.7) activity was determined by [54] with minor adjustments. The reaction mixture comprised of 50 mM KH_2_PO_4_ buffer (pH 7.0), 1% guaiacol (C_7_H_8_O_2_), 0.5% H_2_O_2_ and 100 μL enzyme extract. The alterations owing to guaiacol were estimated at 470 nm. Catalase (CAT, EC 1.11.1.6) was determined by [55] with the use of H_2_O_2_ (extinction co-efficient 39.4 mM cm^− 1^). Glutathione reductase (GR, EC 1.6.4.2) activity was assayed by following the method of [56] with NADPH oxidation at 340 nm (extinction coefficient 6.2 mM cm^− 1^). The assay for ascorbate peroxidase (APX, EC 1.11.1.11) activity was measured by [57] with slight changes. The alterations in reaction mixture were as 100 mM KH_2_PO_4_ buffer (pH 7.0), 0.1 mM EDTA-Na_2_, 0.05 H_2_O_2_, 0.3 mM ascorbic acid, and 100 μL protein extract. The absorbance was checked at 290 nm after 30 s of H_2_O_2_ addition.

### Estimation of thiol compounds and observation of leaf stomata by scanning electron microscopy (SEM)

The estimation of non-protein thiol (NPT), and reduced and oxidized glutathione (GSH and GSSG), respectively) was carried out according to [58]. The concentration of phytochelatins (PCs) was determined as PCs = NPT – (GSH + GSSG) [59]. For SEM, leaf samples were immediately fixed with 2.5% glutaraldehyde and then postfixed with 1% OsO_4_ in (0.1 M) phosphate-buffered saline (PBS; pH 6.8) to evade any damage during sample preparation. The fixed leaves were dehydrated in a graded ethanol solution, transferred to alcohol + iso-amyl acetate (1:1, v/v) mixture, and then transferred to pure iso-amyl acetate. In the end, samples were vacuum-dried in Hitachi Model HCP-2 with liquid C0_2_ and coated with gold-palladium in Hitachi Model E-1010 ion sputter. The SEM observations were made with an S-4800 microscope (Hitachi Led., Tokyo, Japan, Model TM-1000).

### Extraction of total RNA and quantitative real-time PCR (qRT-PCR) assays

Total RNA from leaf and root (about 100 mg) tissues was excerpted manually by a Trizol method. To eliminate the genomic DNA (gDNA) and cDNA synthesis, we used Prime scriptTM RT reagent with gDNA eraser kit (Takara, Co. Ltd., Japan). The synthesized cDNA from different treatment was assayed for quantitative real-time (qRT-PCR) in the iCycler iQTM Real-time detection system (Bio-Rad, Hercules, CA, USA) by using SYBR® Premix Ex Taq II (Takara, Co. Ltd., Japan). Primers for targeted phosphate/silicon genes were obtained from the sequence database of NCBI (http://www.ncbi.nlm.nih.gov). The sequence (5′ → 3′) of forward (F) and reverse (R) primers are given in Additional file 1: Table S2. The PCR conditions were established by adopting the method of [60].

### Statistical analysis

The significant differences were investigated among the physio-biochemical, osmolytes and phytochelatins data. The results represent the mean ± standard deviation of four to six (minimum three) replicates. Data was analyzed by using statistical package, SPSS version 16 (Chicago, IL, USA). A two-way variance analysis (ANOVA) was used followed by Duncan’s Multiple Range Test (DMRT) (*P* < 0.05). For supplementary data, two-way ANOVA and β-coefficients were used followed by Duncan’s Multiple Range Test (DMRT) with significances at *P*, 0.05 and 0.01 [61]. The graphs were prepared by plotting data in Origin Pro version 8.0 (Origin Lab Corporation, Wellesley Hills, Wellesley, MA, USA).

## Results

### Se-induced endogenous MT biosynthesis and exogenous MT reduce se uptake in plant tissues

To determine the effects of exogenous selenium (Se) on endogenous melatonin (MT) biosynthesis and Se uptake, we measured endogenous MT and Se accumulation in *B. napus* leaves and roots at various Se doses (Additional file 1: Table S1). For the control, there were non-significant (*P* ≥ 0.05) differences between the leaf and root in terms of Se content. Substantial increases in leaf and root Se content with increasing Se dose (50 μM, 100 μM, and 200 μM) were observed relative to the controls. Maximum increases in Se content were measured at 200 μM Se. The accumulation in the roots was 1367.21 mg kg^− 1^ DW and in the leaves it was 285.60 mg kg^− 1^ DW. Endogenous MT content and MT induction also increased with Se dose. In contrast, the MT concentrations remained nearly constant in the leaves and roots under non-stress conditions. The Se-treated plants displayed maximum MT biosynthesis at 200 μM Se. At this dosage, the MT levels in the leaves and roots were 59 and 65% and 76 and 85% higher than those at the 50 μM Se and 100 μM Se dosages, respectively. These findings confirmed that exogenous Se induces endogenous MT accumulation in *B. napus* tissues. Selenium accumulation was significantly (*P* ≤ 0.05) more enhanced in the roots than the leaves with increasing Se doses (Additional file 1: Table S1). This phenomenon implies reduced Se translocation to the leaves and greater Se accumulation in the roots. Exogenous MT reduced Se uptake and translocation in plant tissues. Relative to the control, the 100 μM MT treatment significantly (*P* ≤ 0.05) reduced plant Se content by 58 and 61%, 33 and 34%, and 21 and 22% in the leaves and roots at 50 μM, 100 μM, and 200 μM Se, respectively. These findings confirmed that exogenous Se induces the accumulation of endogenous Se and that exogenous MT + Se application reduces Se accumulation in *B. napus* leaves and roots.

### Exogenous MT alleviates se-induced plant growth, biomass accumulation, and photosynthesis reductions

**Fig. 1:**
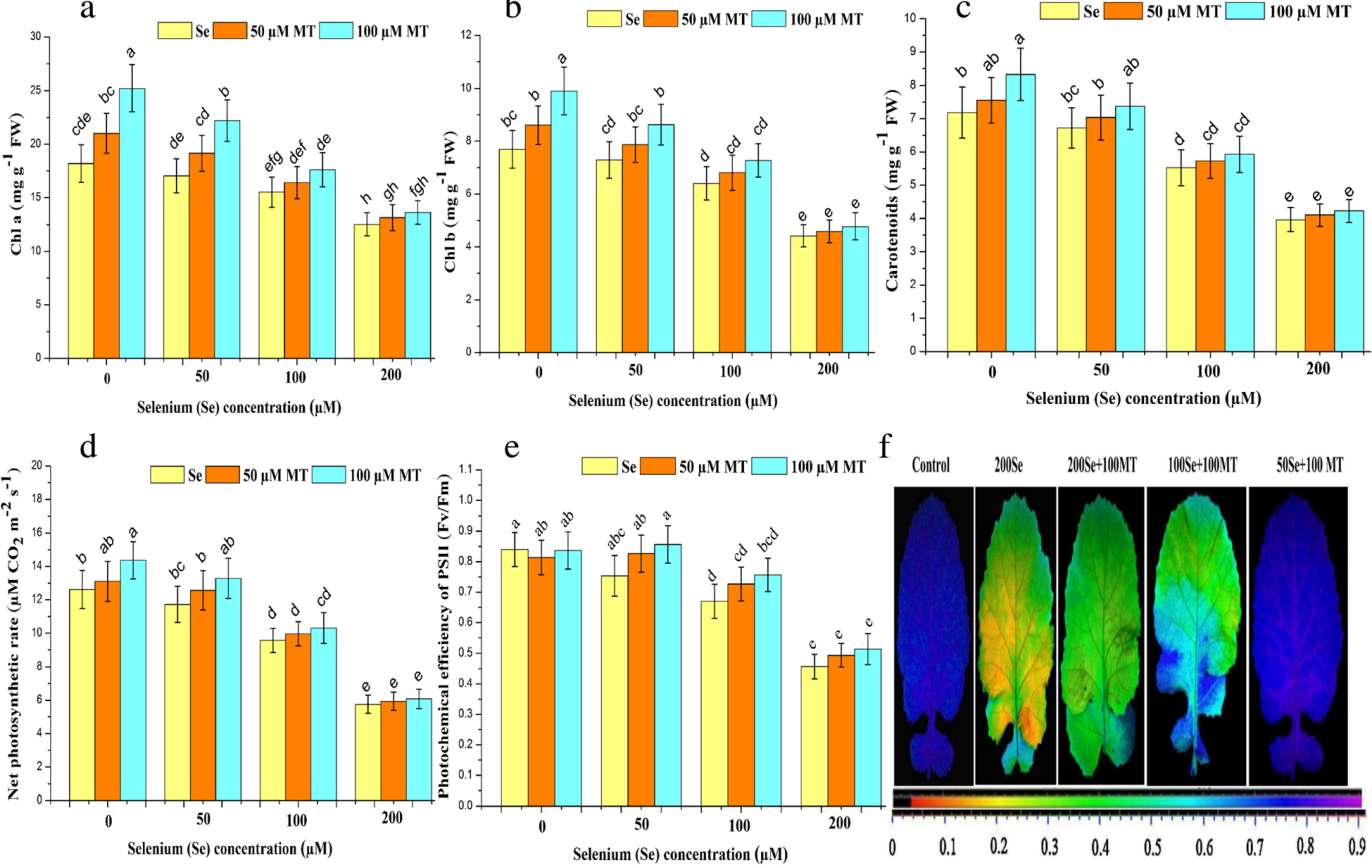
Interactive effects of exogenous melatonin and selenium on photosynthesis traits. Effects of different treatments of exogenous melatonin (MT) (0 μM, 50 μM and 100 μM) and selenium (Se) (0 μM, 50 μM, 100 μM and 200 μM) on the **a**) *Chl a* (mg/g FW), **b**) *Chl b* (mg/g FW), **c**) carotenoids (mg/g FW), **d**) net photosynthetic rate (μM CO_2_ m^− 2^ s^− 1^) and (**e** and **f**) photochemical efficiency of PSII (*Fv/Fm*) of fully stretched leaves of *Brassica napus* cv. ZS 758

Endogenous MT production in *B. napus* seedlings under Se stress suggests that MT participates in biochemical and physiological processes in the plant (Additional file 1: Table S1). We focused on Se-induced phenotypic changes in plant growth, biomass production (Table 1), and photosynthesis (Fig. 1a-f) in order to elucidate the mechanism by which MT mitigates Se stress. For the control, there were no significant differences (*P* ≥ 0.05) between MT level and Se concentration. Selenium at 50 μM caused no significant changes in plant morpho-physiology whereas 100 μM Se slightly modified these attributes of *B. napus*. On the other hand, 200 μM Se induced severe foliar chlorosis and significantly (*P* ≤ 0.05) reduced leaf fresh and dry biomass (49 and 46%), root fresh and dry biomass (39 and 53%), plant height (44%), leaf area (32%), *Chl a* (31%), *Chl b* (43%), carotenoids (45%), net photosynthetic rate (54%), and *Fv/Fm* (46%) relative to the control. All doses of exogenous MT reversed the deleterious effects of Se. The 100 μM MT + 50 μM Se treatment dramatically increased leaf fresh and dry weight (8 and 17%), root fresh and dry weight (25 and 17%), plant height (7%), leaf area (6%), *Chl a* (30%), *Chl b* (18%), carotenoids (10%), net photosynthetic rate (13%), and *Fv/Fm* (14%) compared with the other MT + Se combination treatments. Exogenous MT at 100 μM was more efficacious than 50 μM MT at attenuating the adverse effects of Se stress. Exogenous MT also mitigated growth inhibition in *B. napus* seedlings under Se stress.

**Table 1:** Interactive effects of exogenous melatonin and selenium on plant morphological characteristics. Effects of different treatments of exogenous MT (0 μM, 50 μM and 100 μM) and Se (Se) (0 μM, 50 μM, 100 μM and 200 μM) on the leaf fresh/dry weight (g), root fresh/dry weight (g), plant height (cm) and leaf area (cm^2^ plant^− 1^) of *Brassica napus* cv. ZS 758

MT conc. (μM)	Se conc. (μM)	Leaf fresh weight	Leaf dry weight	Root fresh Weight	Root dry weight	Plant height	Leaf area
0	0	114.59 ± 8.01ab	7.49 ± 0.72ab	16.61 ± 1.55 cd	3.65 ± 0.35ab	25.68 ± 2.43ab	192.22 ± 17.17abc
	50	107.75 ± 7.17b	7.04 ± 0.69bc	15.24 ± 1.38de	3.35 ± 0.33bc	24.61 ± 2.40bcd	185.73 ± 16.57abc
	100	91.08 ± 6.20c	5.84 ± 0.55d	13.51 ± 1.26ef	2.77 ± 0.22d	20.76 ± 2.21c	168.66 ± 14.58c
	200	58.76 ± 4.76d	4.07 ± 0.39e	10.16 ± 0.92 g	1.73 ± 0.17e	14.36 ± 1.68d	130.36 ± 11.64d
50	0	116.03 ± 8.51ab	7.71 ± 0.75ab	17.69 ± 1.55bc	3.77 ± 0.34ab	26.26 ± 2.56a	193.88 ± 17.09abc
	50	110.18 ± 7.88ab	7.58 ± 0.73ab	16.61 ± 1.35 cd	3.59 ± 0.36ab	25.09 ± 2.32ab	188.98 ± 17.24abc
	100	92.27 ± 6.11c	6.25 ± 0.57 cd	14.68 ± 1.21de	2.91 ± 0.24 cd	21.08 ± 2.01c	171.28 ± 15.88bc
	200	59.46 ± 4.20d	4.34 ± 0.42e	10.99 ± 0.89 g	1.80 ± 0.16e	14.55 ± 1.42d	132.11 ± 12.22d
100	0	121.03 ± 8.87a	8.28 ± 0.77a	20.53 ± 1.84a	4.04 ± 0.38a	28.74 ± 2.73a	198.01 ± 17.25a
	50	115.90 ± 7.88ab	8.26 ± 0.80a	19.09 ± 1.79ab	3.92 ± 0.39a	26.25 ± 2.55a	195.96 ± 16.76ab
	100	94.56 ± 5.84c	6.69 ± 0.63bcd	16.50 ± 1.31 cd	3.07 ± 0.25 cd	21.79 ± 2.08bc	175.08 ± 15.01abc
	200	60.75 ± 4.02d	4.59 ± 0.41e	12.11 ± 1.09 fg	1.86 ± 0.15e	14.80 ± 1.47d	134.24 ± 11.84d

Values are means ± St. Dev. (*n* = 3). Means of values followed by the same letters are not significantly differing at *P* ≤ 0.05 according to Duncan’s multiple range test

### Exogenous MT improves metabolic compensation, mitigates oxidative damage, and maintains membrane integrity by reducing se stress

**Fig. 2:**
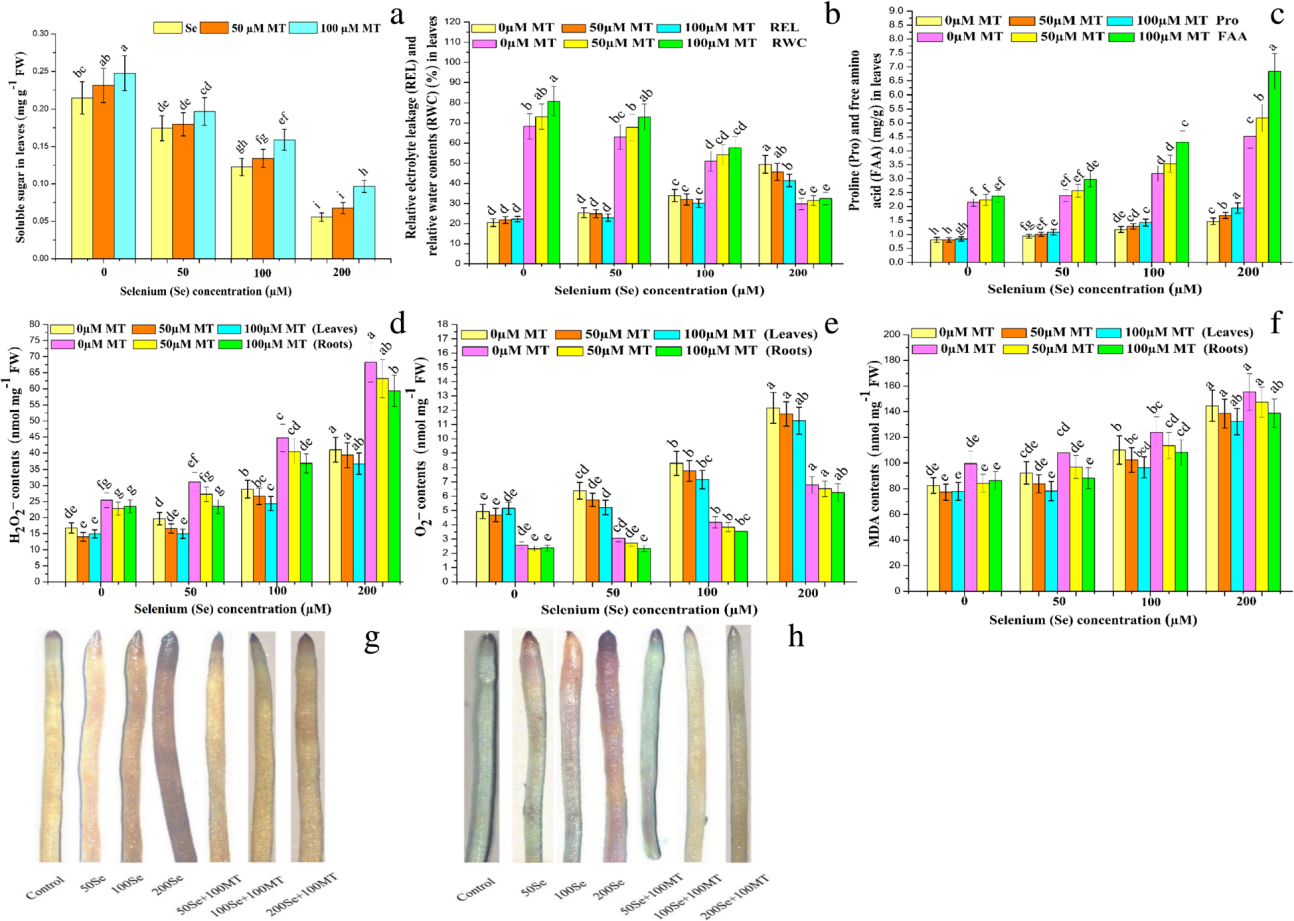
Interactive effects of exogenous melatonin and selenium on osmotic metabolites, reactive oxidative species, relative electrolyte leakage, and histochemical staining. Effects of different treatments of exogenous melatonin (MT) (0 μM, 50 μM and 100 μM) and selenium (Se) (0 μM, 50 μM, 100 μM and 200 μM) on the (**a**) soluble sugar (mg/g FW), (**b**) relative electrolyte leakage (%) and relative water content (%), (**c**) proline contents (mg/g FW) and free amino acid (mg/g FW) in the leaves, and (**d**) H_2_O_2_ (nmol mg^− 1^ FW), (**e**) O_2_^•–^ (nmol mg^− 1^ FW), (**f**) MDA (nmol mg− 1 FW) contents in the leaves and roots, and root staining with (**g**) 3,3-diaminobenzidine (DAB) and (**h**) nitro-blue tetrazolium (NBT) of *Brassica napus* cv. ZS 758

To investigate the role of MT in Se-induced osmotic stress, we compared the relative water content (RWC), water-soluble sugar (WSG), free amino acid (FAA), and proline (Fig. 2a-c) levels among treatments. RWC and WSG decreased with increasing Se dose. The strongest reductions in RWC and WSG occurred at 200 μM Se and they were significant (*P* ≤ 0.05). At this Se dosage, RWC and WSG were 56 and 74% lower than the they were in the control. Exogenously applied MT augmented the RWC and WSG diminished by Se exposure. Maximum RWG and WSG recovery was observed for the 50 μM Se treatment (16%) and minimum recovery was detected for the 200 μM Se treatment (9%) (Fig. 2a and b). The standalone Se treatment significantly (*P* ≤ 0.05) increased FAA and proline compared with the control. The 50 μM, 100 μM, and 200 μM Se doses increased FAA and proline by 11 and 16%, 48 and 46%, and 109 and 82%, respectively. Exogenously applied MT increased foliar FAA and proline with increasing Se dose. Maximum increases in FAA and proline were detected at 100 μM MT + 200 μM Se (51 and 32% higher, respectively, than the other MT + Se treatments). The standalone MT treatment slightly increased foliar FAA and proline relative to the control (Fig. 2c).

The main plant biomarkers of oxidative damage, H_2_O_2_ and O_2_^•–^, were measured in the leaves and roots of *B. napus* under Se stress. Moreover, the roles of MT in alleviating Se-induced oxidative injury were also evaluated (Fig. 2d and e). Considerably more H_2_O_2_ and O_2_^•–^ accumulated in the roots than the leaves. Relative to the control, there were 17 and 22%, 71 and 76%, and 144 and 168% increases in H_2_O_2_ and 29 and 20%, 68 and 63%, and 147 and 165% increases in O_2_^•–^ in the leaves and roots at 50 μM, 100 μM, and 200 μM Se, respectively. Exogenous MT alleviated Se-induced oxidative damage. The strongest MT-mediated reduction in oxidative injury was observed for the 100 μM MT + 50 μM Se treatment wherein the leaf and root H_2_O_2_ and O_2_^•–^ levels were 24 and 25% and 19 and 24% lower, respectively, in comparison with all other MT + Se treatments. To confirm that ROS (H_2_O_2_ and O_2_^•–^) accumulated in the plants under Se stress and that MT attenuated this effect, we stained the roots of *B. napus* plants with 3,3-diaminobenzidine (DAB) and nitro blue tetrazolium (NBT). Compared with the control, the roots of the plants subjected to 200 μM Se presented with dark brown (H_2_O_2_) and dark blue (O_2_^•–^) staining (Fig. 2g and h). In contrast, MT treatment reduced the intensity of the DAB and NBT staining in *B. napus* roots exposed to SE stress. Furthermore, the application of exogenous MT promoted the biosynthesis of endogenous MT (Additional file 1: Table S1). Exogenous MT 100 μM strongly induced endogenous MT accumulation under Se stress.

To investigate the efficacy of exogenous MT at maintaining plasma membrane stability in response to Se stress, we measured malondialdehyde (MDA) and relative electrolyte leakage (REL) (Fig. 2b and f). Relative to the control, there were no significant changes (*P* ≥ 0.05) in REL or MDA in the standalone Se or MT treatments. However, compared with the control, MDA and REL significantly (*P* ≤ 0.05) increased by 11 and 8%, 33 and 24%, and 75 and 56% in the leaves and roots and by 24, 66, and 142% in the leaves at 50 μM, 100 μM, and 200 μM, respectively. Moreover, exogenous MT suppressed increases in MDA and REL relative to the control and the other MT treatments (Fig. 2b and f).

### MT enhances se tolerance by inducing antioxidant enzymes and regulating phosphate/silicon transporters

**Fig. 3:**
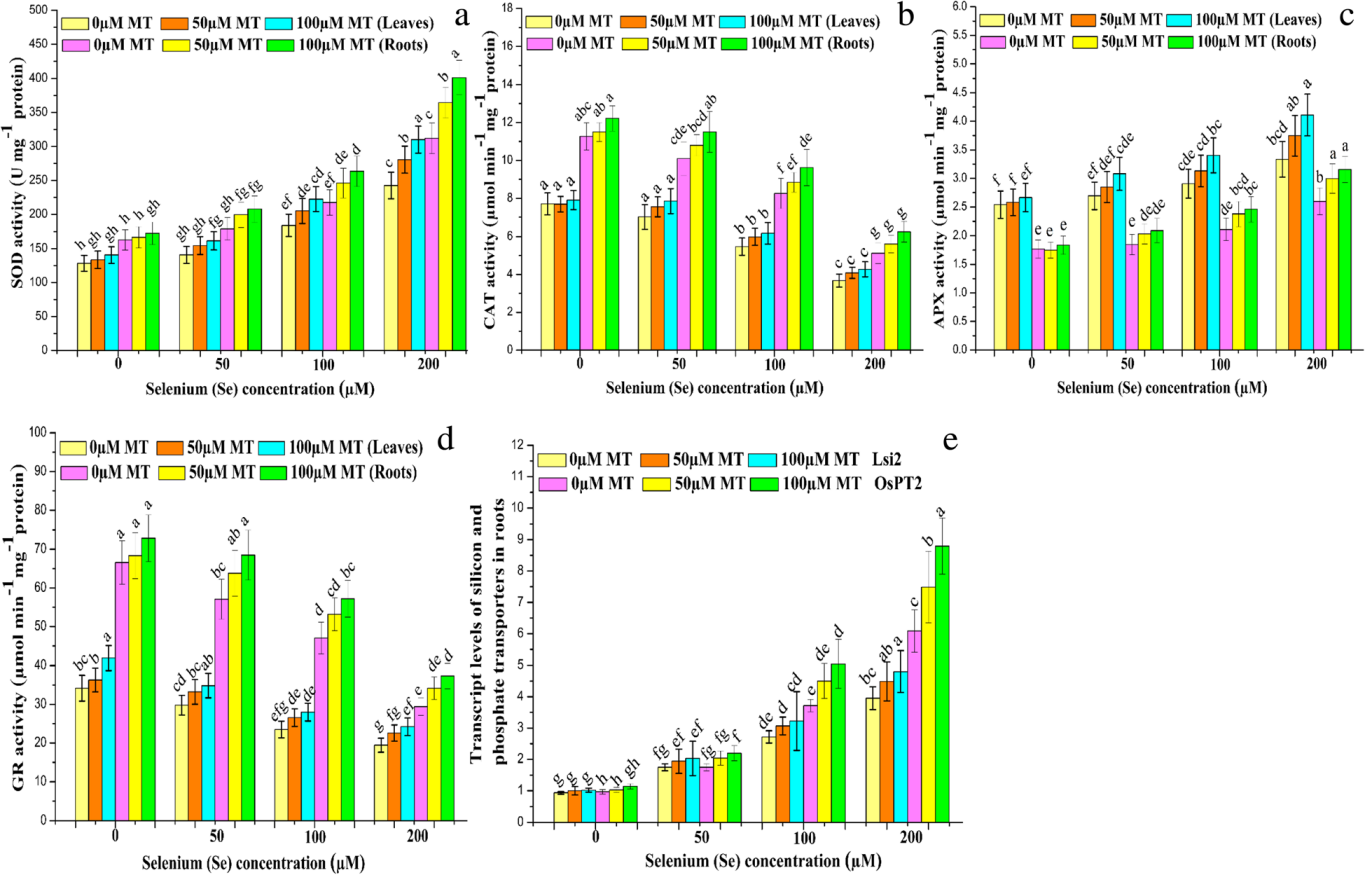
Interactive effects of exogenous melatonin and selenium on the enzyme activities and phosphate/silicon transporters. Effects of different treatments of exogenous melatonin (MT) (0 μM, 50 μM and 100 μM) and selenium (Se) (0 μM, 50 μM, 100 μM and 200 μM) on the activities of (**a**) superoxide dismutase (SOD), (**b**) catalase (CAT), (APX) ascorbate peroxidase (APX), and (**d**) glutathione reductase (GR) in the leaves and roots, and (**e**) phosphate (*OsPT2*)/silicon influx (*Lis*2) transporters in the roots of *Brassica napus* cv. ZS 758

To examine the efficacy of MT at regulating the ROS-scavenging system under Se stress, we measured superoxide dismutase (SOD), ascorbate peroxidase (APX), glutathione reductase (GR), and catalase (CAT) activity in *B. napus* leaves and roots (Fig. 3a-d). SOD and APX activity increased and CAT and GR activity decreased with increasing Se dose. However, the most significant (*P* ≤ 0.05) alterations in antioxidant enzyme activity were detected at 200 μM Se. Exogenous MT further changed the enzyme activity levels under Se stress especially at MT and Se doses of 100 μM and 200 μM, respectively (Fig. 3a-d).

Earlier studies reported that Se (IV) is transportedmainly via phosphate/silicon influx transporters [60, 61]. To evaluate the activity levels of these transporter genes in *B. napus*, we performed an expression analysis on its roots. The gene expression analysis of phosphate and silicon influx transporter (*OsPT2* and *Lis*2) displayed a substantial up-regulation in their gene expressions. The expression of *OsPT2* was more abundant and highly expressed than *Lis*2. These results suggested that *OsPT2* more actively participate in selenite uptake in comparison with *Lis*2 (Fig. 3e). Exogenous MT upregulated both transporter genes but especially *Lis*2 at 100 μM MT + 200 μM Se (IV) (Fig. 3e).

### Exogenous MT stimulates se sequestration by inducing endogenous chelating compounds and their metabolic enzymes

**Fig. 4:**
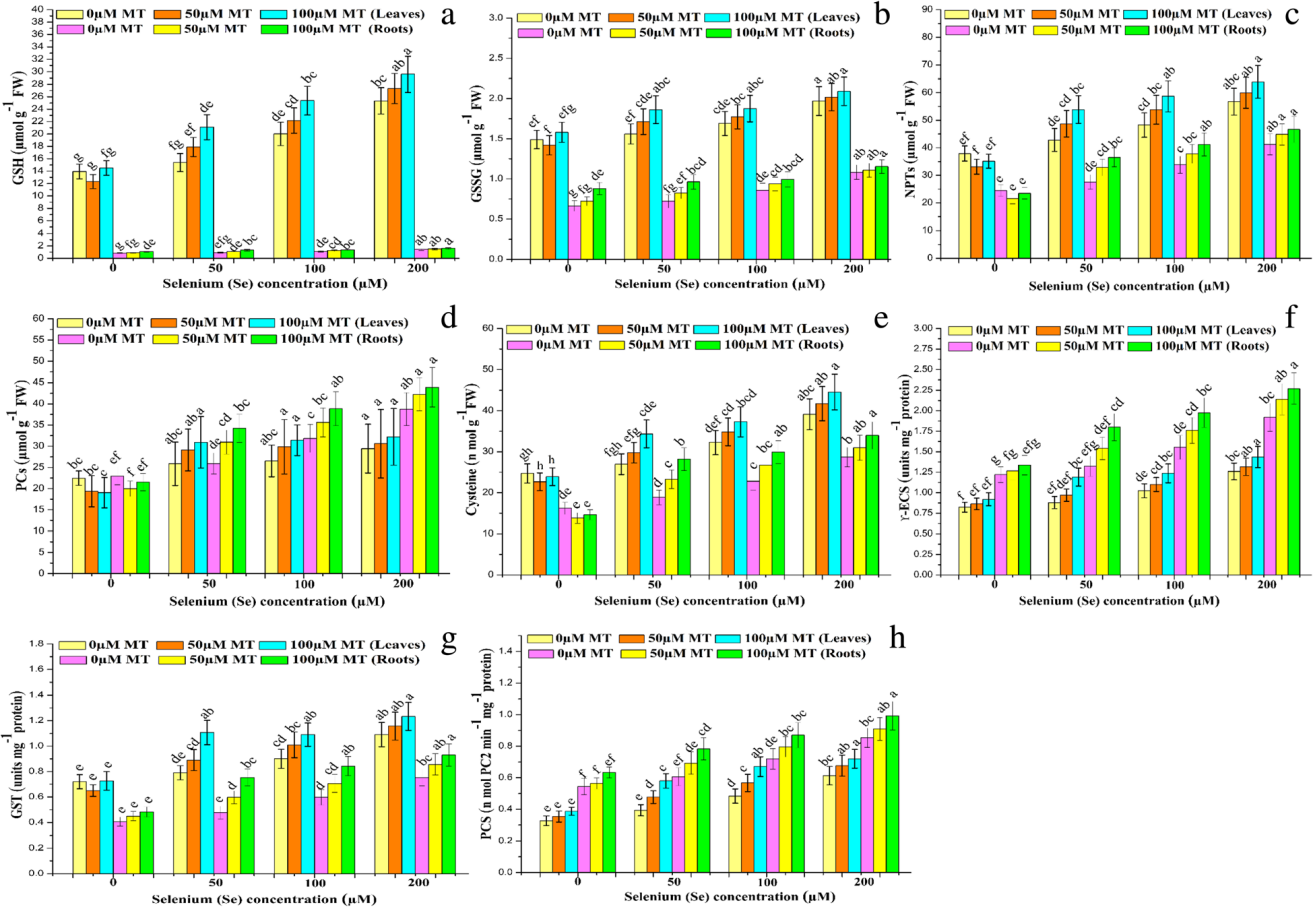
Interactive effects of exogenous melatonin and selenium on the biosynthesis of thiolic components and their metabolic enzymes. Effects of different treatments of exogenous melatonin (MT) (0 μM, 50 μM and 100 μM) and selenium (Se) (0 μM, 50 μM, 100 μM and 200 μM) on the (**a**) reduced glutathione content (GSH) (μmol g^− 1^ FW), (**b**) oxidized glutathione content (GSSG) (μmol g^− 1^ FW), (**c**) non-protein thiols (NPTs) (μmol g^− 1^ FW), (**d**) phytochelatins (PCs) (μmol g^− 1^ FW), (**e**) cysteine (Cyst) (nmol g^− 1^ FW), and (**f**) activities of γ-glutamylcysteine synthetase (γ-ECS) (units mg^− 1^ protein), (**g**) glutathione-S-transferase (GST) (units mg^− 1^ protein) and (**h**) phytochelatins synthase (PCS) (nmol PC2 min^− 1^ mg^− 1^ protein) in the leaves and roots of *Brassica napus* cv. ZS 758

To evaluate the efficacy of MT at inducing chelating agent biosynthesis, we measured the levels of reduced glutathione (GSH), oxidized glutathione (GSSG), non-protein thiols (NPTs), phytochelatins (PCs), and cysteine in the leaves and roots of *B. napus* plants under Se stress (Fig. 4a-e). Standalone Se treatments significantly increased all thiols relative to the control. Maximum increases in thiol content were detected for the 200 μM Se treatment. Exogenous MT further increased thiol levels under Se stress. Compared with the control, maximum increases in thiol content were observed at 100 μM MT + 50 μM Se (37 and 42%, 19 and 34%, 16 and 6%, 26 and 33%, 20 and 32%, and 27 and 49% for GSH, GSSG, GSH/GSSG, NPTs, PCs, and cysteine in the leaves and roots, respectively. To assess the importance of MT in Se detoxification, we measured the enzymes participating in plant thiol metabolism (Fig. 4f-h). Relative to the control, the standalone Se treatment significantly (*P* ≤ 0.05) upregulated gamma-glutamylcysteine synthase (γ-ECS), glutathione-*S*-transferase (GST), and phytochelatin synthase (PCS) by 53 and 57%, 51 and 85%, and 88 and 57% in the leaves and roots, respectively. Exogenous MT further raised the levels of these enzymes. The maximum increases at 100 μM MT + 50 μM Se were 35 and 36% (γ-ECS), 40 and 58% (GST), and 47 and 29% (PCS) in the leaves and roots, respectively, relative to the other MT + Se treatments and the standalone Se treatment. The observed increases in thiol metabolism in the MT + Se treatments compared with the standalone Se treatment suggested that MT participates in Se detoxification.

### Exogenous MT facilitates stomatal opening

**Fig. 5:**
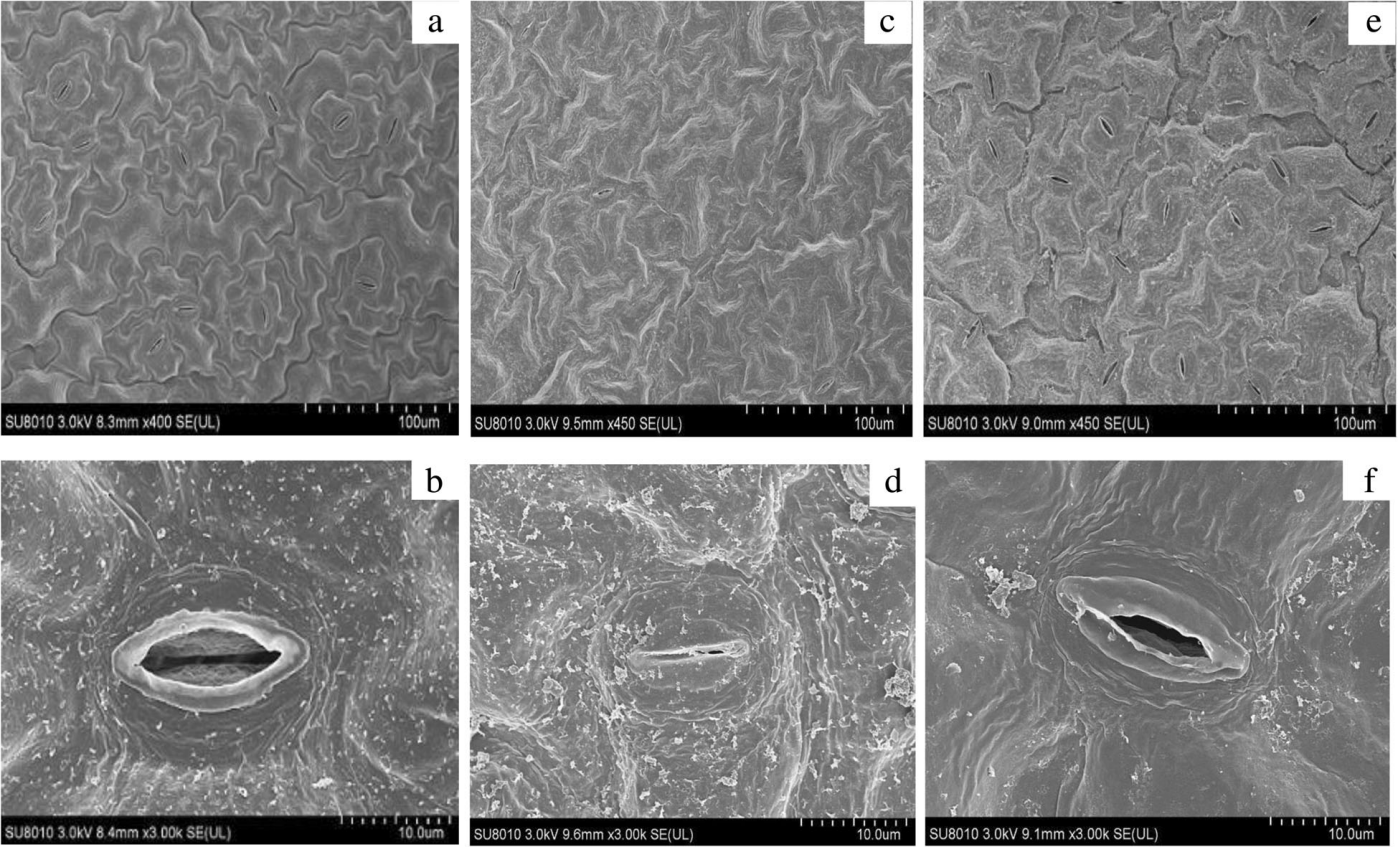
Interactive effects of exogenous melatonin and selenium on stomatal opening. Scanning electron microscope (SEM) images of stomata showed the responses of exogenous MT on the stomatal aperture of *Brassica napus* leaves under Se stress. **a** and **b** showing full opening of leaves stomata under no stress conditions. **c** and **d** showed the complete closure of leaves stomata under maximum Se (200 μM) stress conditions. **e** and **f** illustrated the maximum stomatal opening at 50 μM Se + 100 μM MT than other Se + MT treatments

Scanning electron microscopy (SEM) disclosed that stomatal length and width were smaller in the leaves of *B. napus* subjected to Se stress than those of the control. However, Se exposure had no apparent effect on stomatal movement (Fig. 5a-e). The stomata of *B. napus* leaves treated with exogenous MT were longer, wider, and more open than those of *B. napus* plants subjected to Se stress alone. MT may facilitate stomatal opening by osmotically retaining water in the leaves. Interactions among the levels of selenium, melatonin, and all aforementioned parameters were evaluated by two-way ANOVA and a β-regression model (Additional file 1: Tables S3-S10).

## Discussion

Selenium (Se) phytotoxicity is a major concern for agricultural scientists [9–11]. As sessile organisms, plants have developed multifaceted strategies to contend with various stressors. Recently, certain researchers and scientists have investigated the use of the growth regulator melatonin (MT) to increase plant resistance to cold [20], salt [21], drought [22], and cadmium [23] stress.

The efficacy of exogenous MT against plant stress depends upon the mode of application (pretreatment/foliar spray/nutrient solution), dosage, stressor, and plant species. It was reported that foliar MT spray (25 μM, 50 μM, and 100 μM) against Cd stress (25 μM and 100 μM) enhanced plant growth and antioxidant systems by inhibiting Cd accumulation [12]. However, MT seed soak/root immersion/foliar spray (20 μM and 100 μM) reduced cold-induced oxidative damage by upregulating the enzymatic/non-enzymatic antioxidant systems [62]. Exogenous MT applied as a nutrient solution (50 μM and 200 μM) improved growth, biomass, and the antioxidant systems of *Cyphomandra betacea* [34] and wheat seedlings [63] under Cd stress.

The results of this study showed that exogenously applied Se increases endogenous MT in plant tissues (Additional file 1: Table S1). This finding corroborated those of earlier reports in which Se pretreatment induced endogenous MT while exogenous MT + Se application reduced growth retardation and photoinhibition in tomato plants [36]. Se-induced MT biosynthesis may promote Se tolerance in *B. napus*. The observed increases in MT content with Se level indicate that MT biosynthesis could be induced by oxidative stress and/or other associated mechanisms. Here, exogenous MT induced de novo endogenous MT production (Additional file 1: Table S1) as it did in rice [64] and wheat [63]. Thus, exogenous MT increased the endogenous MT content and may regulate the antioxidant system and restrict ROS generation. In turn, de novo endogenous MT production in *B. napus* may help alleviate Se phytotoxicity by mitigating Se-induced oxidative damage.

Plant biomass decreased with increasing Se dosage. Exogenous MT recovered the reduction in biomass accumulation caused by Se stress (Table 1). Se-induced declines in plant growth and biomass accumulation were observed in rice [9] possibly as a result of chlorophyll damage and protein synthesis inhibition. Exogenous MT (50 μM and 100 μM) alleviated Cd stress and promoted growth and biomass formation in *Cyphomandra betacea* [4] and wheat [60], respectively. In the present study, MT attenuated Se-induced chlorophyll degradation and improved photosynthetic efficiency under both non-stress and Se-stress conditions (Fig. 1a-f). Previous reports revealed that MT repressed chlorophyll degradation and enhanced photosynthetic efficiency in cucumber [65], wheat [66], gardenia [67], and tomato [12, 68] under water, heat, low-light, cadmium, and cold stresses, respectively. MT might maintain chlorophyll and carotenoid levels by scavenging excessive ROS [12]. The declines in net photosynthetic rate and photochemical efficiency (*Fv/Fm*) under Se stress (Fig. 1e and f) caused photoinhibition. Therefore, stress-induced ROS production reduces PSII photochemical efficiency by interrupting the electron transport chain (ETC) [69]. In contrast, exogenous MT significantly alleviated photoinhibition and increased photosynthetic efficiency via biostimulant pathways that enhanced PSII photochemical efficiency [70]. Exogenous MT application restrained the decline in PSII efficiency in response to Se stress by making the maximum amount of light energy available to the photosynthetic ETC. Se-induced reduction in the photosynthetic rate may trigger stomatal closing (Fig. 5a-f). Deformation of the guard cells may be caused by inhibition of the metabolic reactions maintaining guard cell turgor. MT increased stomatal length and width by keeping the water potential (Fig. 2b) and proline (Fig. 2c) levels high, thereby opening the stomata (Fig. 5e and f). MT maintained cell turgor, increased proline levels, and opened stomata under drought stress [71]. In the present study, exogenous MT effectively recovered the Se-induced decline in the water-holding capacity of *B. napus* leaves (Fig. 2b). Previous reports demonstrated that exogenous MT mitigated water losses in wheat [72] and maize [73] under salt stress. In tomato leaves under drought conditions, MT recovered foliar water losses by promoting an increase in cuticular wax thickness [74]. Therefore, MT may protect plants against water stress by elevating foliar water potential, minimizing water losses, and maintaining plant metabolism.

Proline, sugars, and free amino acids are biocompatible solutes that protect plants against stress conditions by osmoregulation, ROS scavenging, and plasma membrane integrity maintenance [75]. Here, exogenous MT increased the Se-induced rises in the proline and free amino acid levels and recovered the reduction in soluble sugar content (Fig. 3a-c). Plants usually restore osmotic equilibrium by accumulating excess osmolytes such as proline [76]. The observed increases in proline level in Se-stressed plants treated with exogenous MT (Fig. 2b) reflect the ability of *B. napus* leaves to contend with oxidative damage [77]. Compatible solutes scavenge excess ROS. Exogenous MT increased the proline content in gardenia plants under dark-induced stress [67]. Under plant stress, then, MT may regulate proline metabolism via antioxidant mechanisms. The observed decline in sugar accumulation in plants under Se stress (Fig. 2a) may be explained by protein deformation resulting from the substitution of selenium for sulfur in S-containing proteins such as cysteine and methionine [78]. MT may have effectively repaired this damage (Fig. 2b). It was recently shown that MT recovered protein damage in *B. napus* leaves under salt stress by increasing their soluble sugar content [79]. The additional increases in free amino acids in response to MT application to plants subjected to Se stress (Fig. 2c) suggests that MT induced protein hydrolysis and osmotic adjustments under these conditions. It was also indicated that the application of the growth regulator 5-aminolevulinic acid (5-ALA) adjusts plant metabolism by inducing foliar free amino acid accumulation in *B. napus* and maintaining or restoring protein structural integrity [80]. The observed marked increases in electrolyte leakage, H_2_O_2_ and O_2_^•–^, and MDA in *B. napus* under Se stress (Fig. 2b, d-f) caused severe oxidative damage and lipid peroxidation and the loss of plasma membrane integrity. Exogenous MT reversed Se-induced oxidative damage by reducing ROS and MDA content and decreasing electrolyte leakage.

Previous studies disclosed that exogenous MT maintained oxidative homeostasis by reducing ROS and MDA accumulation as well as electrolyte leakage in tomato [62] and rice [64] subjected to cold stress. Earlier reports demonstrated that exogenous MT activates antioxidant enzymes and promotes the accumulation of non-enzymatic antioxidants to offset damage caused by environmental stressors [22, 63]. Exogenous MT upregulated SOD, APX, GR, and CAT which, in turn, scavenged the excess ROS produced under Se stress (Fig. 3a-d). Therefore, exogenous MT may act as signaling molecule that induces the antioxidant defense system and diminishes the Se-induced oxidative damages.

Previous studies documented that Se is either transported by sulfate or phosphate transporter genes [6, 40, 41]. In current study, selenite was suggested to be mediated mainly by phosphate transporters rather than silicon influx transporters (Fig. 3e) which revealed the key role of phosphate transport pathway in the uptake of selenite. Although further convincing molecular evidence is required to support this investigation. This hypothesis was strongly supported by previous results that selenite uptake was more pronounced in both wild type and mutant plants under phosphate-deficient conditions which resulted in the activation of phosphate transporters to enhance the phosphate uptake. And, concluded that phosphate transporters are directly involved in selenite uptake [6, 40].

Various enzymatic pathways synthesize phytochelatins from GSH via metal and metalloid ion chelation [81]. The thiols cysteine, GSH, GSSG, NPTs, and PCs participate in metalloid detoxification [82]. The present study revealed that plants under Se stress accumulate comparatively high levels of thiols. Moreover, exogenous MT further increases plant thiol levels (Fig. 4a-e). Thus, plants attempt to detoxify Se by increasing thiol content. MT-induced thiol biosynthesis sequestered and detoxified Cd in tomato [12]. Here, MT enhanced GR activity (Fig. 3d) and increased the GSH:GSSG ratio (Additional file 1: Table S3) in *B. napus.* These effects could induce γ-ECS (Fig. 4f). The observed increase in GSH (Fig. 4a) caused by MT may increase γ-ECS activity which, in turn, delays leaf senescence (Fig. 4f). These responses were also reported for apple trees [70]. In another study, exogenous MT upregulated *SIGSH1* and *SIPCS* in tomato leaves. These genes encode GSH and PCs, respectively [12]. The thiol-metabolizing enzymes γ-ECS, GST, and PCS participated mostly in GSH biosynthesis and conjugation, respectively [83]. Here, thiol-metabolizing enzymes were induced in response to plant Se exposure possibly in the attempt to detoxify it. MT further augmented this mechanism (Fig. 4f-h). The observed upregulation of thiol-metabolizing enzymes in response to de novo thiol biosynthesis induced by Se exposure was accompanied by an increase in NPTs (mainly GSH and PCs) (Fig. 4a, c, and d). Previous studies stated that arsenic (As) stress upregulated thiol-metabolizing enzymes as well as NPTs [14]. Here, relative to the standalone Se treatment, the MT + Se treatments induced greater accumulations of cysteine (Fig. 4e) and GSH (Fig. 4a). This response may enable plants to increase sulfur metabolism and mediate thiol metabolism for Se detoxification. Elevated cysteine and GSH levels could improve sulfur metabolism which, in turn, may detoxify arsenic [84]. The relatively higher levels of thiols in plants under the MT + Se treatment than in those exposed to Se alone indicate that MT is very effective at Se detoxification. The comparatively greater accumulation of chelating compounds such as PCs in the roots suggested that these organs are major brunt of Se detoxification. In addition, the augmented thiols accumulation in roots than leaves of MT-treated *B. napus* suggests that MT more effectively sequesters Se in the roots and lowers its mobility so that it is not readily translocated to the leaves (Additional file 1: Table S1). Previous studies proposed that MT may prevent Cd translocation from root to leaf possibly by enhancing de novo thiol biosynthesis [12].

## Conclusions

**Fig. 6:**
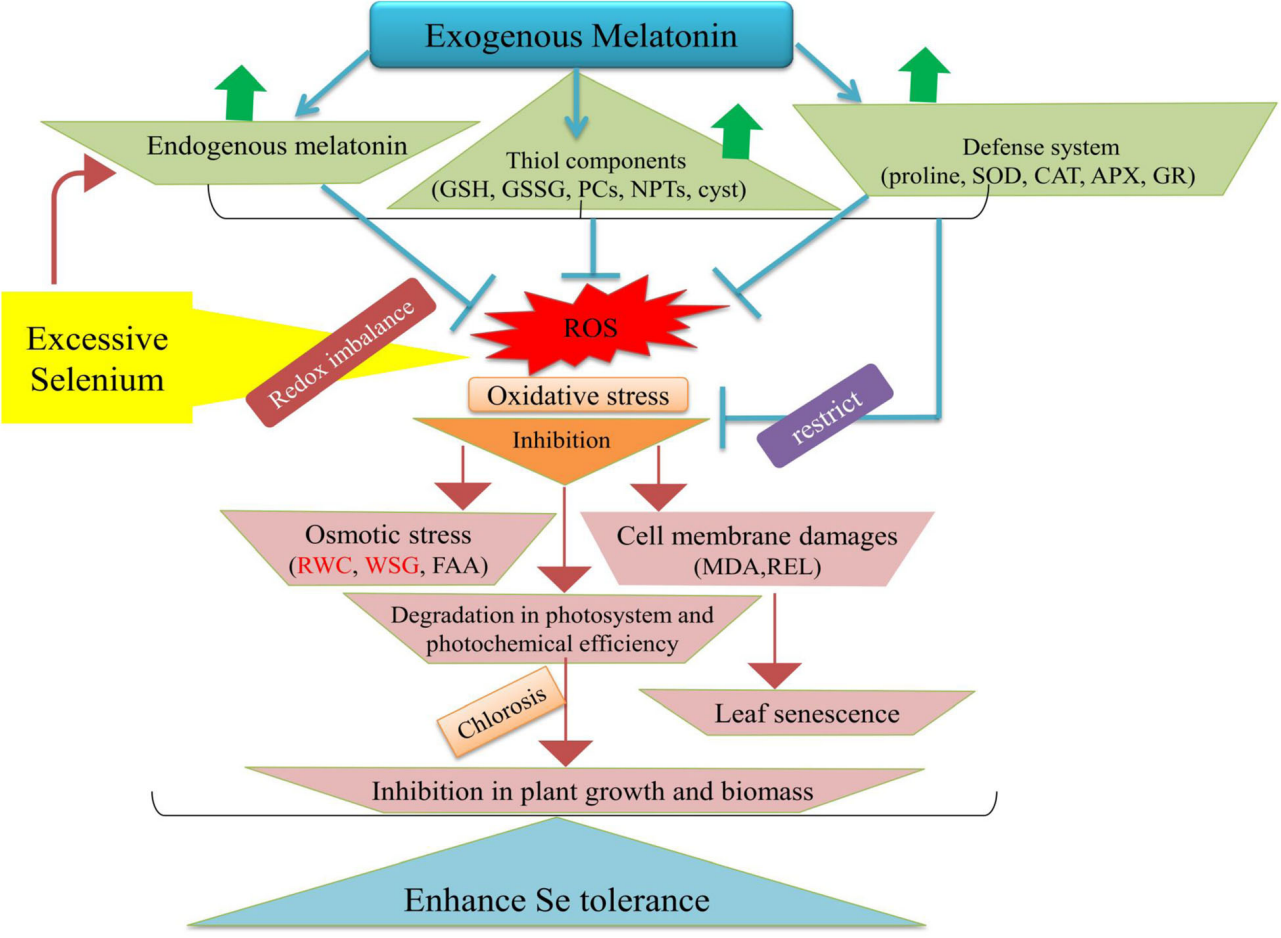
Summary of protective mechanisms of melatonin against selenium phytotoxicity. A schematic diagram showed the mitigating effects of exogenous MT on *Brassica napus* L. seedlings under Se (IV) stress. Se heightened its toxicity by (I) Over-accumulating ROS that leads to chlorophyll degradation and ultimately growth reduction. (II) Induction of electrolyte leakage and lipid peroxidation reflects the damages in cellular membrane. (III) Disturbances in the synchronization of the defense system by increasing SOD and APX activities, proline, and free amino acids but declined the key enzymes (CAT and GR) and soluble sugar. (IV) Osmotic stress by lowering relative water and sugar contents. (V) An increase in the levels of thiol compounds (GSH, GSSG, NPTs, cysteine, and PCs) depicted the greater potential of *Brassica napus* plants to confer Se tolerance. Exogenous MT ameliorated the Se toxicity by enhancing photochemical efficiency and osmo-protection, which is linked with the enhanced plant growth and biomass production. In addition, exogenous MT induced the endogenous MT content which assist in the protective role of MT against Se-prompted ROS generation by inducing enzymes involved in AsA-GSH cycle (APX and GR), ROS-detoxifying enzymes (mainly SOD and CAT), biosynthesis of thiol components (especially GSH and phytochelatins), and the enzymes involved in thiol metabolism (γ-ECS, GST and PCS). The greater accumulation of MT and thiol components in roots suggested roots as greater site for the detoxification of Se as compared with leaves. Diagram indicates O_2_^•–^ (superoxide), H_2_O_2_ (hydrogen peroxide), SOD (superoxide dismutase), CAT (catalase), APX (ascorbate peroxidase), GR (glutathione reductase), GSH (reduced glutathione), GSSG (oxidized glutathione), RWC (relative water content), Pro (proline), WSG (water soluble sugar), FAA (free amino acids), REL (relative electrolyte leakage), MDA (melondialdehyde), NPTs (non-protein thiols), PCs (phytochelatins), cyst (cysteine), γ-ECS (gamma-glutamylcysteine synthase), GST (glutathione-S-transferase) and PCS (phytochelatins synthase)

Based on our findings, a schematic diagram was plotted to highlight the Se-induced toxic effects in *Brassica napus* plants mitigated by exogenous MT (Fig. 6). Here, we confirmed that high Se concentrations reduced plant growth and biomass production, impaired PSII photochemical efficiency (*Fv/Fm*), decreased *Chl a*, *Chl b*, and carotenoid levels, lowered the net photosynthetic rate, increased osmotic stress by decreasing RWC, and altered stomata size and shape. Selenium also destroyed plasma membrane integrity by promoting lipid peroxidation and oxidative damage. These effects were reflected in the observed increases in REL, MDA, H_2_O_2_, and O_2_^•–^ levels. Elevated Se perturbed the plant antioxidant system by enhancing SOD and APX activity and increasing proline and FAA levels and chelator biosynthesis. However, reduced the CAT and GR activity and soluble sugar concentrations. Co-application of exogenous MT and excess Se induced de novo endogenous MT production. MT also increased antioxidant enzyme activity, scavenged excess ROS, improved photosynthetic capacity, restored water levels, and protected plasma membranes against lipid peroxidation. Exogenous MT increased RWC, decreased photoinhibition, and lowered the REL and MDA levels. Thus, exogenous MT enhances plant growth and biomass accumulation under Se stress. It also augmented plant oxidative stress defense and Se detoxification by inducing the antioxidant system and enhancing the Se binding capacity of GSH, GSSG, NPTs, PCs, and cysteine. In the present study, 100 μM exogenous MT was the most efficacious dose for protecting *B. napus* plants against the toxic effects of Se. Our findings demonstrate that exogenous MT improves Se tolerance and minimized the Se-accumulation in *B. napus* plants. These findings provide implications in understanding the effect of plant MT and develop strategies for safe food production in Se-enriched soils. However, the molecular mechanisms, genetic evidences and signaling pathways by which exogenous MT mediates Se detoxification and induces MT biosynthesis merit further exploration. Further studies are recommended in soil-based environment by using other application methods (foliar spray and seed priming with MT) to reveal the possible plant-protection against other environmental pollutants such as cobalt, beryllium, nickel, and strontium. Our future study will be focused on the identification of molecular networks of MT in the regulation of abiotic stresses in *B. napus*.

## Supplementary information

**Additional file 1: Table S1.** Effects of exogenous melatonin (MT) (0 μM, 50 μM and 100 μM) and selenium (Se) (0 μM, 50 μM, 100 μM, and 200 μM) treatments on the endogenous MT and Se contents in the leaves and roots of *Brassica napus* cv. ZS 758**. Table S2.** Oligonucleotide primer sequences, used for qRT-PCR analysis. **Table S3.** Effects of different treatments of melatonin (MT) (0 μM, 50 μM and 100 μM) and selenium (Se) (0 μM, 50 μM, 100 μM, and 200 μM) on the ratio of GSH/GSSG (μM/g FW) in the leaves and roots of *Brassica napus* cv. ZS 758. **Table S4.** Two-way ANOVA and multiple regression model for the morphological traits of *Brassica napus* cv. ZS 758. **Table S5.** Two-way ANOVA and multiple regression model for the photosynthesis traits of *Brassica napus* cv. ZS 758. **Table S6.** Two-way ANOVA and multiple regression model for the osmotic metabolites in the leaves of *Brassica napus* cv. ZS 758. **Table S7.** Two-way ANOVA and multiple regression model for the reactive oxygen species (ROS) and malondialdehyde (MDA) contents in the leaves and roots of *Brassica napus* cv. ZS 758. **Table S8.** Two-way ANOVA and multiple regression model for the antioxidant enzymes (μmol minr^− 1^ mg^− 1^ protein) in the leaves and roots of *Brassica napus* cv. ZS 758. **Table S9.** Two-way ANOVA and regression analysis for the thiol components in the leaves (L) and roots (R) of *Brassica napus* cv. ZS 758. **Table S10.** Two-way ANOVA and regression analysis for the thiolic ligands related metabolic enzymes and endogenous selenium (Se) contents in the leaves (L) and roots (R) of *Brassica napus* cv. ZS 758.

## Data Availability

The datasets used and/or analyzed during the current study available from the corresponding author on reasonable request.

## Abbreviations

APX: Ascorbate peroxidase
CAT: Catalase
cyst: cysteine
FAA: Free amino acids
GR: Glutathione reductase
GSH: Reduced glutathione
GSSG: Oxidized glutathione
GST: Glutathione-S-transferase
H_2_O_2_: Hydrogen peroxide
MDA: Melondialdehyde
NPTs: Non-protein thiols
O_2_: Superoxide radical
PCs: Phytochelatins
PCS: Phytochelatins synthase
Pro: Proline
REL: Relative electrolyte leakage
RWC: Relative water content
SOD: Superoxide dismutase
WSG: Water-soluble sugar
γ-ECS: Gamma-glutamylcysteine synthase
