# Supplementary material for: Protective mechanisms of melatonin against selenium toxicity in Brassica napus: insights into physiological traits, thiol biosynthesis and antioxidant machinery
Source: BMC Plant Biol. 2019 Nov 21;19:507. doi: 10.1186/s12870-019-2110-6 (PMC6869276; doi:10.1186/s12870-019-2110-6)
Supplement: Supplementary file 1 — Additional file 1: Table S1. Effects of exogenous melatonin (MT) (0 μM, 50 μM and 100 μM) and selenium (Se) (0 μM, 50 μM, 100 μM, and 200 μM) treatments on the endogenous MT and Se contents in the leaves and roots of Brassica napus cv. ZS 758. Table S2. Oligonucleotide primer sequences, used for qRT-PCR analysis. Table S3. Effects of different treatments of melatonin (MT) (0 μM, 50 μM and 100 μM) and selenium (Se) (0 μM, 50 μM, 100 μM, and 200 μM) on the ratio of GSH/GSSG (μM/g FW) in the leaves and roots of Brassica napus cv. ZS 758. Table S4. Two-way ANOVA and multiple regression model for the morphological traits of Brassica napus cv. ZS 758. Table S5. Two-way ANOVA and multiple regression model for the photosynthesis traits of Brassica napus cv. ZS 758. Table S6. Two-way ANOVA and multiple regression model for the osmotic metabolites in the leaves of Brassica napus cv. ZS 758. Table S7. Two-way ANOVA and multiple regression model for the reactive oxygen species (ROS) and malondialdehyde (MDA) contents in the leaves and roots of Brassica napus cv. ZS 758. Table S8. Two-way ANOVA and multiple regression model for the antioxidant enzymes (μmol minr− 1 mg− 1 protein) in the leaves and roots of Brassica napus cv. ZS 758. Table S9. Two-way ANOVA and regression analysis for the thiol components in the leaves (L) and roots (R) of Brassica napus cv. ZS 758. Table S10. Two-way ANOVA and regression analysis for the thiolic ligands related metabolic enzymes and endogenous selenium (Se) contents in the leaves (L) and roots (R) of Brassica napus cv. ZS 758. [file 12870_2019_2110_MOESM1_ESM.docx]

**Table S1** Effects of exogenous melatonin (MT) (0, 50 and 100 µM) and selenium (Se) (0, 50, 100, and 200 µM) treatments on the endogenous MT and Se contents in the leaves and roots of *Brassica napus* cv. ZS 758.

| Exogenous Se conc.  (µM) | Exogenous MT conc.  (µM) | Endogenous Me content  (ng/g FW)    Leaf Root | | Endogenous Se content  (mg/kg DW)    Leaf Root | |
| --- | --- | --- | --- | --- | --- |
| 0 | 0 | 79.47±6.24g | 83.54±5.86f | 0.04±0.02j | 0.06±0.03j |
|  | 50 | 92.57±7.12fg | 112.43±8.49e | 0.16±0.03j | 0.15±0.03j |
|  | 100 | 130.74±11.6de | 143.01±13.65d | 0.21±0.04j | 0.16±0.03j |
| 50 | 0 | 93.26±5.53fg | 94.45±8.25ef | 91.08±5.49g | 244.49±15.22g |
|  | 50 | 110.92±9.24ef | 143.75±9.96d | 58.20±4.99h | 145.53±13.87h |
|  | 100 | 167.78±11.82c | 194.75±17.76c | 37.91±3.47i | 94.94±12.93i |
| 100 | 0 | 111.56±6.61ef | 103.33±8.01ef | 170.16±11.37d | 632.90±22.26d |
|  | 50 | 126.91±11.23e | 159.87±11.80d | 134.68±9.23e | 489.75±18.51e |
|  | 100 | 199.49±13.90b | 224.10±18.32b | 114.17±10.40f | 418.22±27.76f |
| 200 | 0 | 124.54±9.70e | 116.00±9.63e | 285.60±16.08a | 1367.21±28.01a |
|  | 50 | 147.08±12.59d | 185.54±11.73c | 250.80±14.93b | 1183.83±26.05b |
|  | 100 | 230.75±17.39a | 264.43±18.86a | 225.51±14.74c | 1061.53±34.41c |

Values are means ± St. Dev. (n=3). Means of values followed by same letters are not significantly differ at P≤ 0.05 according to Duncan’s multiple range test.

**Table S2** Oligonucleotide primer sequences, used for qRT-PCR analysis.

| Gene name | Forward | Reverse |
| --- | --- | --- |
| OsPT2 | GGGAGAGAGCTCTAATGGCG | GGGATTGGATCTGGGTGTGG |
| OsNIP2;1 (*Lis*2) | GAAGACGAGCAGCGAGTAGG | GCGGAAGCTGTTCCTCAAGA |
| Actin | TTGGGATGGACCAGAAGG | TTGGGATGGACCAGAAGG |

**Table S3** Effects of different treatments of melatonin (MT) (0, 50 and 100 µM) and selenium (Se) (0, 50, 100, and 200 µM) on the ratio of GSH/GSSG (µM/g FW) in the leaves and roots of *Brassica napus* cv. ZS 758.

| Melatonin conc.  (µM) | Se. conc. (µM) | GSH/GSSG ratio (µM/g FW)  Leaf Root | |
| --- | --- | --- | --- |
| 0 | 0 | 9.44±1.53ef | 1.27±0.18a |
|  | 50 | 9.86±0.15def | 1.28±0.03a |
|  | 100 | 11.95±2.04abcd | 1.29±0.05a |
|  | 200 | 12.91±1.25ab | 1.31±0.25a |
| 50 | 0 | 8.68±0.52f | 1.24±0.10a |
|  | 50 | 10.51±1.33cdef | 1.33±0.03a |
|  | 100 | 12.60±1.88abc | 1.33±0.23a |
|  | 200 | 13.52±0.17ab | 1.35±0.22a |
| 100 | 0 | 9.26±1.5ef | 1.22±0.19a |
|  | 50 | 11.43±1.9bcde | 1.36±0.14 |
|  | 100 | 13.53±0.52ab | 1.37±0.03a |
|  | 200 | 14.21±1.65a | 1.38±0.10a |

Values are means ± St. Dev. (n=3). Means of values followed by same letters are not significantly differ at P≤ 0.05 according to Duncan’s multiple range test.

**Table S4** Two-way ANOVA and multiple regression model for the morphological characteristics of *Brassica napus* cv. ZS 758.

| Source *(df)* | *F-ratios* | | | |
| --- | --- | --- | --- | --- |
|  | Leaf area Plant height Leaf dry weight  (cm^2^) (cm) (g) | Leaf fresh weight  (g) | Root dry weight (g) | Root fresh weight  (g) |
| Cv. (2, 36) | 0.562 1.605 5.358* | 1.724 | 4.339* | 16.481*** |
| Conc. (3, 36) | 30.655** 56.607*** 58.171*** | 130.112*** | 88.812*** | 47.00*** |
| Cv.*Conc. (6, 36) | 0.025 0.230 0.178 | 0.138 | 0.299 | 0.391 |

Cv. cultivar, Conc. concentration **p* < 0.05, ***p* < 0.01 and ****p* < 0.001, respectively.

| Multiple linear regression model | $\beta$-Regression coefficients | Multiple correlation coefficient $(r)$ |
| --- | --- | --- |
| Plant length = 27.386+0. 772$X_{1}$-4.107$X_{2}$ | $\beta_{1}$ (Cv.) = 0.122 | 0.900*** |
|  | $\beta_{2}$ (Conc.) = -0. 892 |  |
| Leaf fresh weight = 121.386+2.508$X_{1}$-19.131$X_{2}$ | $\beta_{1}$ (Cv.) = 0. 088 | 0.925*** |
|  | $\beta_{2}$ (Conc.) = -0. 921 |  |
| Root fresh weight = 17.271+1.587$X_{1}$-2.365$X_{2}$ | $\beta_{1}$ (Cv.) = 0.400 | 0.908*** |
|  | $\beta_{2}$ (Conc.) = -0. 816 |  |
| Leaf dry weight = 7.866+0. 422$X_{1}$-1.184$X_{2}$ | $\beta_{1}$ (Cv.) = 0.225 | 0.894** |
|  | $\beta_{2}$ (Conc.) = -0. 865 |  |
| Root dry weight = 3.881+0.174$X_{1}$-0.678$X_{2}$ | $\beta_{1}$ (Cv.) = 0.169 | 0.916*** |
|  | $\beta_{2}$ (Conc.) = -0.900 |  |
| Leaf area = 199.813+3.290$X_{1}$-20.596$X_{2}$ | $\beta_{1}$ (Cv.) = 0.097 | 0.833** |
|  | $\beta_{2}$ (Conc.) = -0. 828 |  |
| *X*_1_ cultivar, *X*_2_ concentration, Cv. cultivar, Conc. concentration **p* < 0.05, ***p* < 0.01 and ****p* < 0.001, respectively |  |  |

**Table S5** Two-way ANOVA and multiple regression model for the photosynthesis traits of *Brassica napus* cv. ZS 758.

| Source *(df)* | *F-ratios* | | | | |  |
| --- | --- | --- | --- | --- | --- | --- |
|  | Chl a Chl b Carotenoids  (mg g^−1^ FW) (mg g^−1^ FW) (mg g^−1^ FW) | Net photosynthetic rate  (µM CO_2_ m^-2^ s^-1^) | | Photochemical efficiency of PSII (Fv/Fm) |  | |
| Cv. (2, 36) | 17.226*** 9.869*** 3.256 | 4.011* | 3.654* | |  | |
| Conc. (3, 36) | 46.196*** 66.332*** 64.744*** | 111.740*** | 72.795*** | |  | |
| Cv. *Conc. (6, 36) | 2.209 1.074 0.350 | 0.425 | 0.677 | |  | |

Cv. cultivar, Conc. concentration **p* < 0.05, ***p* < 0.01 and ****p* < 0.001, respectively.

| Multiple linear regression model | $\beta$-Regression coefficients | Multiple correlation coefficient $(r)$ |
| --- | --- | --- |
| Chl a = 19.926+1.923$X_{1}$-2.812$X_{2}$ | $\beta_{1}$ (Cv.) = 0.403 | 0.902*** |
|  | $\beta_{2}$ (Conc.) = -0. 807 |  |
| Chl b = 8.451+0.598$X_{1}$-1.352$X_{2}$ | $\beta_{1}$ (Cv.) = 0.281 | 0.912*** |
|  | $\beta_{2}$ (Conc.) = -0.868 |  |
| Carotenoids = 7.648+0. 307$X_{1}$-1.210$X_{2}$ | $\beta_{1}$ (Cv.) = 0.169 | 0.926*** |
|  | $\beta_{2}$ (Conc.) = -0. 910 |  |
| Net photosynthetic rate = 13.624+0.547$X_{1}$-2.489$X_{2}$ | $\beta_{1}$ (Cv.) = 0.147 | 0.927*** |
|  | $\beta_{2}$ (Conc.) = -0. 915 |  |
| Fv/Fm = 0.850+0.030$X_{1}$-0.112$X_{2}$ | $\beta_{1}$ (Cv.) = 0.169 | 0.870** |
|  | $\beta_{2}$ (Conc.) = -0. 853 |  |
| *X*_1_ cultivar, *X*_2_ concentration, Cv. cultivar, Conc. concentration **p* < 0.05, ***p* < 0.01 and ****p* < 0.001, respectively |  |  |

**Table S6** Two-way ANOVA and multiple regression model for the osmotic metabolites in the leaves of *Brassica napus* cv. ZS 758.

| Source *(df)* | *F-ratios* | | | |
| --- | --- | --- | --- | --- |
|  | Relative water content Soluble sugar Relative electrolyte leakage  (%) (mg/g FW) (%) | Free amino acid  (mg/g FW) | Proline  (mg/g FW) |  |
| Cv. (2, 36) | 6.272** 13.572*** 3.985* | 29.319*** | 13.452*** |  |
| Conc. (3, 36) | 108.445*** 161.821*** 138.246*** | 158.051*** | 114.588*** |  |
| Cv.*Conc. (6, 36) | 0.472 0.245 1.654 | 5.593** | 2.315 |  |

Cv. cultivar, Conc. concentration **p* < 0.05, ***p* < 0.01 and ****p* < 0.001, respectively.

| Multiple linear regression model | $\beta$-Regression coefficients | Multiple correlation coefficient $(r)$ |
| --- | --- | --- |
| Relative water content = 74.270+3.952$X_{1}$-14.223$X_{2}$ | $\beta_{1}$ (Cv.) = 0.185 | 0.931*** |
|  | $\beta_{2}$ (Conc.) = -0. 913 |  |
| Soluble sugar = 0.218+0.017$X_{1}$-0.052$X_{2}$ | $\beta_{1}$ (Cv.) = 0.221 | 0.972*** |
|  | $\beta_{2}$ (Conc.) = -0.946 |  |
| Relative electrolyte leakage = = 20.475-1.565$X_{1}$+7.971$X_{2}$ | $\beta_{1}$ (Cv.) = -0.131 | 0.923*** |
|  | $\beta_{2}$ (Conc.) = 0.914 |  |
| Free amino acids = 1.374+0.529$X_{1}$+1.081$X_{2}$ | $\beta_{1}$ (Cv.) = 0.307 | 0.912*** |
|  | $\beta_{2}$ (Conc.) = 0.859 |  |
| Proline = 0.652+0.114$X_{1}$+0.294$X_{2}$ | $\beta_{1}$ (Cv.) = 0.256 | 0.942*** |
|  | $\beta_{2}$ (Conc.) = 0. 906 |  |
| *X*_1_ cultivar, *X*_2_ concentration, Cv. cultivar, Conc. concentration  **p* < 0.05, ***p* < 0.01 and ****p* < 0.001, respectively |  |  |

**Table S7** Two-way ANOVA and multiple regression model for the reactive oxygen species (ROS) and melondialdehyde (MDA) contents in the leaves and roots of *Brassica napus* cv. ZS 758

| Source *(df)* | *F-ratios* | | |  |
| --- | --- | --- | --- | --- |
|  | MDA in leaves MDA in roots O_2_ in leaves O_2_ in roots    (nmol mg^-1^ FW) (nmol mg^-1^ FW) | H_2_O_2_ in leaves H_2_O_2_ in roots    (nmol mg^-1^ FW) | |  |
| Cv. (2, 36) | 4.700* 7.805 3.404 5.807** | 7.404** | 9.223** |  |
| Conc. (3, 36) | 79.667*** 54.814*** 167.447*** 220.536*** | 177.601*** | 204.534*** |  |
| Cv.*Conc.(6, 36) | 0.203 0.200 0.724 0.336 | 0.392 | 0.552 |  |

Cv. cultivar, Conc. concentration **p* < 0.05, ***p* < 0.01 and ****p* < 0.001, respectively.

| Multiple linear regression model | $\beta$-Regression coefficients | Multiple correlation coefficient $(r)$ |
| --- | --- | --- |
| MDA in leaves = 27.386+0. 772$X_{1}$-4.107$X_{2}$ | $\beta_{1}$ (Cv.) = 0.122 | 0.900*** |
|  | $\beta_{2}$ (Conc.) = -0. 892 |  |
| MDA in roots = 121.386+2.508$X_{1}$-19.131$X_{2}$ | $\beta_{1}$ (Cv.) = 0. 088 | 0.925*** |
|  | $\beta_{2}$ (Conc.) = -0. 921 |  |
| O_2_ in leaves = 17.271+1.587$X_{1}$-2.365$X_{2}$ | $\beta_{1}$ (Cv.) = 0.400 | 0.908*** |
|  | $\beta_{2}$ (Conc.) = -0. 816 |  |
| O_2_ in roots = 7.866+0. 422$X_{1}$-1.184$X_{2}$ | $\beta_{1}$ (Cv.) = 0.225 | 0.894** |
|  | $\beta_{2}$ (Conc.) = -0. 865 |  |
| H_2_O_2_ in leaves = 3.881+0.174$X_{1}$-0.678$X_{2}$ | $\beta_{1}$ (Cv.) = 0.169 | 0.916*** |
|  | $\beta_{2}$ (Conc.) = -0.900 |  |
| H_2_O_2_ in roots = 199.813+3.290$X_{1}$-20.596$X_{2}$ | $\beta_{1}$ (Cv.) = 0.097 | 0.833** |
|  | $\beta_{2}$ (Conc.) = -0. 828 |  |
| *X*_1_ cultivar, *X*_2_ concentration, Cv. cultivar, Conc. concentration  **p* < 0.05, ***p* < 0.01 and ****p* < 0.001, respectively |  |  |

**Table S8** Two-way ANOVA and multiple regression model for the antioxidant enzymes (µmol minr^-1^ mg^-1^ protein) in the leaves and roots of *Brassica napus* cv. ZS 758

| Source(df) | | $F-ratios$ | | | | | | | | | | |  | |  |  |
| --- | --- | --- | --- | --- | --- | --- | --- | --- | --- | --- | --- | --- | --- | --- | --- | --- |
|  | | | SOD in  leaves | SOD in  roots | CAT in  leaves | | CAT in  roots | APX in  leaves | | APX in  roots | GR in  leaves | GR in  Roots | |  | |  |
| Cv. (2, 36) | | | 14.233*** | 14.680*** | 4.244** | | 1.75 | 7.285** | | 7.216** | 12.879*** | 10.022** | |  | |  |
| Conc. (3, 36) | | | 145.956*** | 164.384*** | 108.553*** | | 43.54*** | 25.793*** | | 54.116*** | 59.110*** | 91.486*** | |  | |  |
| Cvs.*Conc. (6, 36) | | | 1.779 | 2.255 | 0.283 | | 1.39 | 0.679 | | 0.915 | 0.469 | 0.217 | |  | |  |
|  | | Cv. cultivar, Conc. concentration **p* < 0.05, ***p* < 0.01 and ****p* < 0.001, respectively. | | | | | | | | | | |  | |  |  |
| Multiple linear regression model | | | | | $\beta$-Regression coefficients | | | Multiple correlation coefficient $(r)$ | | | | | | | |  |
| SOD in leaves = 102.301+17.350$X_{1}$+48.221$X_{2}$ | | | | | $\beta_{1}$ (Cv.) = 0.238 | | | 0.936*** | | | | | | | |  |
|  | | | | | $\beta_{2}$ (Conc.) = 0. 905 | | |  | | | | | | | |  |
| SOD in roots = 125.878+21.750$X_{1}$+62.291$X_{2}$ | | | | | $\beta_{1}$ (Cv.) = 0.227 | | | 0.920*** | | | | | | | |  |
|  | | | | | $\beta_{2}$ (Conc.) = 0.891 | | |  | | | | | | | |  |
| CAT in leaves = 7.920+0.293$X_{1}$-1.288$X_{2}$ | | | | | $\beta_{1}$ (Cv.) = 0.152 | | | 0.928*** | | | | | | | |  |
|  | | | | | $\beta_{2}$ (Conc.) = -0.915 | | |  | | | | | | | |  |
| CAT in roots = 11.638+0.606$X_{1}$-1.991$X_{2}$ | | | | | $\beta_{1}$ (Cv.) = 0.204 | | | 0.938*** | | | | | | | |  |
|  | | | | | $\beta_{2}$ (Conc.) = -0.916 | | |  | | | | | | | |  |
| APX in leaves = 2.312+0.223$X_{1}$+0.368$X_{2}$ | | | | | $\beta_{1}$ (Cv.) = 0.348 | | | 0.860** | | | | | | | |  |
|  | | | | | $\beta_{2}$ (Conc.) = 0.787 | | |  | | | | | | | |  |
| APX in roots = 1.540+0.153$X_{1}$+0.372$X_{2}$ | | | | | $\beta_{1}$ (Cv.) = 0.259 | | | 0.900*** | | | | | | | |  |
|  | | | | | $\beta_{2}$ (Conc.) = 0.862 | | |  | | | | | | | |  |
| GR in leaves = 34.692+2.763$X_{1}$-5.275$X_{2}$ | | | | | $\beta_{1}$ (Cv.) = 0.334 | | | 0.936*** | | | | | | | |  |
|  | | | | | $\beta_{2}$ (Conc.) = -0.875 | | |  | | | | | | | |  |
| GR in roots = 67.785+4.472$X_{1}$-11.755$X_{2}$ | | | | | $\beta_{1}$ (Cv.) = 0.250 | | | 0.934*** | | | | | | | |  |
|  | | | | | $\beta_{2}$ (Conc.) = -0.900 | | |  | | | | | | | |  |
| *X*_1_ cultivar, *X*_2_ concentration, Cv. cultivar, Conc. concentration  **p* < 0.05, ***p* < 0.01 and ****p* < 0.001, respectively | | | | |  | | |  | | | | | | | |  |

**Table S9** Two-way ANOVA and regression analysis for the thiol components in the leaves (L) and roots (R) of *Brassica napus* cv. ZS 758

| Source (df) | | $F-ratios$ | | | | | | | | | | |  |  |  |
| --- | --- | --- | --- | --- | --- | --- | --- | --- | --- | --- | --- | --- | --- | --- | --- |
|  | | GSH (µmol g^-1^)  (L) (R) | | GSSG (µmol g^-1^)  (L) (R) | | GSH/GSSG ratio  (L) (R) | | NPTs (µmol g^-1^)  (L) (R) | | PCs (µmol g^-1^)  (L) (R) |  | | | |  |
| Cv. (2, 36) | | 13.307*** | 17.616*** | 4.135* | 12.574*** | 1.955 | 0.229 | 5.996** | 7.328** 0.593 6.397** | |  | | | |  |
| Conc. (3, 36) | | 84.928*** | 43.354*** | 18.415*** | 32.067*** | 19.269*** | 0.821 | 46.492*** | 64.973*** 7.281** 61.774*** | |  | | | |  |
| Cvs.*Conc. (6, 36) | | 1.292 | 0.649 | 0.417 | 0.794 | 0.327 | 0.123 | 1.588 | 1.536 0.471 1.558 | |  | | | |  |
|  | | Cv. cultivar, Conc. concentration **p* < 0.05, ***p* < 0.01 and ****p* < 0.001, respectively. | | | | | | | | | | |  |  |  |
| Multiple linear regression model | | | | | $\beta$-Regression coefficients | | | Multiple correlation coefficient $(r)$ | | |  |  |  |  |  |
| GSH in leaves = 11.554+1.985$X_{1}$+4.585$X_{2}$ | | | | | $\beta_{1}$ (Cv.) = 0.285 | | | 0.946*** | | |  |  |  |  |  |
|  | | | | | $\beta_{2}$ (Conc.) = 0. 902 | | |  | | |  |  |  |  |  |
| GSH in roots = 0.792+0.131$X_{1}$+0.182$X_{2}$ | | | | | $\beta_{1}$ (Cv.) = 0.425 | | | 0.916*** | | |  |  |  |  |  |
|  | | | | | $\beta_{2}$ (Conc.) = 0.811 | | |  | | |  |  |  |  |  |
| GSSG in leaves = 1.418+0.088$X_{1}$+0.166$X_{2}$ | | | | | $\beta_{1}$ (Cv.) = 0.296 | | | 0.823** | | |  |  |  |  |  |
|  | | | | | $\beta_{2}$ (Conc.) = 0.768 | | |  | | |  |  |  |  |  |
| GSSG in roots = 0.649+0.083$X_{1}$+1.009$X_{2}$ | | | | | $\beta_{1}$ (Cv.) = 0.406 | | | 0.882** | | |  |  |  |  |  |
|  | | | | | $\beta_{2}$ (Conc.) = 0.783 | | |  | | |  |  |  |  |  |
| GSH/GSSG in leaves = 8.657+0.534$X_{1}$+1.534$X_{2}$ | | | | | $\beta_{1}$ (Cv.) = 0.204 | | | 0.828** | | |  |  |  |  |  |
|  | | | | | $\beta_{2}$ (Conc.) = 0. 803 | | |  | | |  |  |  |  |  |
| GSH/GSSG in roots = 1.247+0.021$X_{1}$+0.033$X_{2}$ | | | | | $\beta_{1}$ (Cv.) = 0.129 | | | 0.302* | | |  |  |  |  |  |
|  | | | | | $\beta_{2}$ (Conc.) = 0.273 | | |  | | |  |  |  |  |  |
| NPTs in leaves = 34.246+3.237$X_{1}$+7.949$X_{2}$ | | | | | $\beta_{1}$ (Cv.) = 0.252 | | | 0.885** | | |  |  |  |  |  |
|  | | | | | $\beta_{2}$ (Conc.) = 0.848 | | |  | | |  |  |  |  |  |
| NPTs in roots = 21.471+2.588$X_{1}$+6.851$X_{2}$ | | | | | $\beta_{1}$ (Cv.) = 0.246 | | | 0.924*** | | |  |  |  |  |  |
| PCs in leaf = 21.275+1.164$X_{1}$+3.198$X_{2}$  PCs in root= 20.030+2.374$X_{1}$+6.552$X_{2}$  Cysteine in leaf = 21.762+2.133$X_{1}$+5.860$X_{2}$  Cysteine in root = 13.664+2.521$X_{1}$+5.208$X_{2}$ | | | | | $\beta_{2}$ (Conc.) = 0.890  $\beta_{1}$ (Cv.) = 0.154  $\beta_{2}$ (Conc.) = 0. 580  $\beta_{1}$ (Cv.) = 0.235  $\beta_{2}$ (Conc.) = 0.888  $\beta_{1}$ (Cv.) = -0.235  $\beta_{2}$ (Conc.) = 0.885  $\beta_{1}$ (Cv.) = 0.302  $\beta_{2}$ (Conc.) = 0.856 | | | 0.600*  0.919***  0.916***  0.907*** | | |  |  |  |  |  |
| *X*_1_ cultivar, *X*_2_ concentration, Cv. cultivar, Conc. concentration **p* < 0.05, ***p* < 0.01 and ****p* < 0.001, respectively | | | | |  | | |  | | |  |  |  |  |  |

**Table S10** Two-way ANOVA and regression analysis for the thiolic ligands related metabolic enzymes and endogenous selenium (Se) contents in the leaves (L) and roots (R) of *Brassica napus* cv. ZS 758

| Source (df) | | | $F-ratios$ | | | | | | | | | |  | | |  |
| --- | --- | --- | --- | --- | --- | --- | --- | --- | --- | --- | --- | --- | --- | --- | --- | --- |
|  | | Cysteine (nmol g^-1^)  (L) (R) | | | ϒ-ECS (units mg^-1^ protein)  (L) (R) | | GST (units mg^-1^ protein)  (L) (R) | | PCS (nmol PC2 min^-1^ mg^-1^ protein)  (L) (R) | | | Se (mg/kg)  (L) (R) | | |  |  |
| Cv. (2, 36) | | 5.485* | | 14.050*** | 14.161*** | 15.163*** | 11.605*** | 29.113* | 23.285*** | | 13.921*** 60.051*** 212.273*** | | | |  |  |
| Conc. (3, 36) | | 50.643*** | | 77.619*** | 40.216*** | 48.318*** | 45.318*** | 66.337*** | 68.591*** | | 44.682*** 1186.880*** 6103.566*** | | | |  |  |
| Cvs.*Conc. (6, 36) | | 0.977 | | 3.174* | 0.736 | 0.856 | 1.931 | 1.586 | 1.290 | | 0.270 6.807*** 31.430*** | | | |  |  |
|  | | | Cv. cultivar, Conc. concentration **p* < 0.05, ***p* < 0.01 and ****p* < 0.001, respectively. | | | | | | | | | |  | | |  |
| Multiple linear regression model | | | | | | $\beta$-Regression coefficients | | | Multiple correlation coefficient $(r)$ | | | |  |  |  |  |
| Cysteine in leaves = 21.762+2.133$X_{1}$+5.860$X_{2}$  Cysteine in roots = 13.664+2.521$X_{1}$+5.208$X_{2}$ | | | | | | $\beta_{1}$ (Cv.) = -0.235 | | | 0.916*** | | | |  |  |  |  |
|  |  |  |  |  |  | $\beta_{2}$ (Conc.) = 0.885 | | |  | | | |  |  |  |  |
|  |  |  |  |  |  | $\beta_{1}$ (Cv.) = 0.302  $\beta_{2}$(Conc.) = 0.856 | | | 0.907*** | | | |  |  |  |  |
| ϒ-ECS in leaves = 0.758+0.100$X_{1}$+0.151$X_{2}$ | | | | | | $\beta_{1}$ (Cv.) = 0.392  $\beta_{2}$ (Conc.) = 0. 816  $\beta_{1}$ (Cv.) = 0.385  $\beta_{2}$ (Conc.) = 0.838  $\beta_{1}$ (Cv.) = 0.338  $\beta_{2}$ (Conc.) = 0.819  $\beta_{1}$ (Cv.) = 0.447  $\beta_{2}$ (Conc.) = 0.825  $\beta_{1}$ (Cv.) = 0.405  $\beta_{2}$ (Conc.) = 0.848 | | |  | | | |  |  |  |  |
|  |  |  |  |  |  |  |  |  | 0.905*** | | | |  |  |  |  |
|  |  |  |  |  |  |  |  |  |  | | | |  |  |  |  |
| ϒ-ECS in roots = 1.097+0.171$X_{1}$+0.272$X_{2}$ | | | | | |  |  |  | 0.923*** | | | |  |  |  |  |
|  | | | | | |  |  |  |  | | | |  |  |  |  |
| GST in leaves = 0.647+0.082$X_{1}$+0.145$X_{2}$ | | | | | |  |  |  | 0.886** | | | |  |  |  |  |
|  | | | | | |  |  |  |  | | | |  |  |  |  |
| GST in roots = 0.361+0.097$X_{1}$+0.131$X_{2}$ | | | | | |  |  |  | 0.938*** | | | |  |  |  |  |
|  | | | | | |  |  |  |  | | | |  |  |  |  |
| PCS in leaves = 0.298+0.068$X_{1}$+0.103$X_{2}$ | | | | | |  |  |  | 0.940*** | | | |  |  |  |  |
|  | | | | | |  |  |  |  | | | |  |  |  |  |
| PCS in roots = 0.510+0.070$X_{1}$+0.112$X_{2}$ | | | | | | $\beta_{1}$ (Cv.) = 0.384 | | | 0.928*** | | | |  |  |  |  |
| Se content in leaf = 9.361-21.135$X_{1}$+83.879$X_{2}$  Se content in root = -39.310-85.309$X_{1}$+395.640$X_{2}$ | | | | | | $\beta_{2}$ (Conc.) = 0.845  $\beta_{1}$ (Cv.) = -0.178  $\beta_{2}$ (Conc.) = 0.965  $\beta_{1}$ (Cv.) = -0.148  $\beta_{2}$ (Conc.) = 0.941 | | | 0.982***  0.953*** | | | |  |  |  |  |
| *X*_1_ cultivar, *X*_2_ concentration, Cv. cultivar, Conc. concentration **p* < 0.05, ***p* < 0.01 and ****p* < 0.001, respectively | | | | | |  | | |  | | | |  |  |  |  |
